# Isolation of T cell receptors targeting recurrent neoantigens in hematological malignancies

**DOI:** 10.1186/s40425-018-0386-y

**Published:** 2018-07-13

**Authors:** Vanessa M. Tubb, Deborah S. Schrikkema, Nathan P. Croft, Anthony W. Purcell, Carsten Linnemann, Manon R. Freriks, Frederick Chen, Heather M. Long, Steven P. Lee, Gavin M. Bendle

**Affiliations:** 1 Institute of Immunology and Immunotherapy, Cancer Immunology and Immunotherapy Centre, University of Birmingham, Birmingham, UK; 2 Infection and Immunity Program and Department of Biochemistry and Molecular Biology, Biomedicine Discovery Institute, Monash University, Clayton, VIC Australia; 3 Division of Immunology, Netherlands Cancer Institute, Amsterdam, Netherlands; 4 Centre for Clinical Haematology, Queen Elizabeth Hospital NHS Foundation Trust, Birmingham, UK

**Keywords:** Neoantigens, TCR gene therapy, CALR, FBXW7, Immunotherapy

## Abstract

Mutation-derived neoantigens represent an important class of tumour-specific, tumour rejection antigens, and are attractive targets for TCR gene therapy of cancer. The majority of such mutations are patient-specific and targeting these requires a fully personalized approach. However, some mutations are found recurrently among cancer patients, and represent potential targets for neoantigen-specific TCR gene therapy that is more widely applicable. Therefore, we have investigated if some cancer mutations found recurrently in hematological malignancies encode immunogenic neoantigens presented by common European Caucasoid HLA class I alleles and can form targets for TCR gene therapy. We initially focused on identifying HLA class I neoepitopes derived from calreticulin (CALR) exon 9 mutations, found in ~ 80% of JAK2wt myeloproliferative neoplasms (MPN). Based on MHC class I peptide predictions, a number of peptides derived from mutant CALR (mCALR) were predicted to bind to HLA-A*03:01 and HLA-B*07:02. However, using mass spectrometry and ex vivo pMHC multimer staining of PBMC from MPN patients with CALR exon 9 mutations, we found no evidence that these peptides were naturally processed and presented on the surface of mCALR-expressing target cells. We next developed a protocol utilizing pMHC multimers to isolate CD8^+^ T cells from healthy human donor PBMC that are specific for mCALR and additional putative neoepitopes found recurrently in hematological malignancies. Using this approach, CD8^+^ T cells specific for HLA-A*03:01- and HLA-B*07:02-presented mCALR peptides and an HLA-A*11:01-presented mutant FBXW7 (mFBXW7) peptide were successfully isolated. TCRs isolated from mCALR-specific CD8^+^ T cell populations were not able to recognize target cells engineered to express mCALR. In contrast, mFBXW7-specific CD8^+^ T cells were able to recognize target cells engineered to express mFBXW7. In conclusion, while we found no evidence for mCALR derived neoepitope presentation in the context of the HLA class I alleles studied, our data suggests that the recurrent pR465H mutation in FBXW7 may encode an HLA-A*11:01 presented neoepitope, and warrants further investigation as a target for T cell based immunotherapy of cancer.

**Electronic supplementary material:**

The online version of this article (10.1186/s40425-018-0386-y) contains supplementary material, which is available to authorized users.

## Background

Non-synonymous somatic mutations can encode neoantigens that are processed and presented by major histocompatibility complexes (MHC) on the surface of mutated tumour cells. Recognition of neoantigens by the endogenous T cell repertoire has been shown to contribute to the therapeutic efficacy of cancer immunotherapy in both mice and humans [1–6]. Therefore, neoantigens represent an important class of tumour-specific, tumour rejection antigens, which can be targeted by neoantigen-specific T cell receptors (TCRs). The majority of such mutations are known to be unique to individual patients and targeting these would thus require a fully personalized approach. However, in addition to these patient-specific mutations, some mutations are found recurrently among particular groups of cancer patients. These shared mutations represent potential targets for a more widely applicable T cell-based immunotherapy.

In 2013, mutations in exon 9 of the calreticulin gene (*CALR*) were discovered in ~ 80% of *JAK2wt* myeloproliferative neoplasm patients (MPN) [7, 8]. Intriguingly, all these exon 9 mutations result in a + 1 bp frameshift resulting in a gain of 36 amino acids. This generates a novel C terminus of the protein that is common to all MPN patients carrying mutations in *CALR* exon 9. Importantly, *CALR* exon 9 mutations were suggested to be early initiating events in MPN, and more recently mutant CALR (mCALR) has been shown to mediate thrombopoietin-independent activation of the thrombopoietin receptor MPL [9, 10]. mCALR is therefore an ideal target for T cell-based immunotherapy given its expression profile and role in driving malignancy.

The genetic engineering of patient T cells with tumour-specific TCRs, known as TCR gene therapy, is a cellular immunotherapeutic approach which aims to rapidly generate a pool of patient-specific tumour-reactive T cells for adoptive transfer. The identification of tumour-specific T cells and isolation of their TCRs represents a bottleneck in the development of TCR gene therapy. However, the healthy donor-derived T cell pool potentially represents a source that can be exploited for the isolation of neoantigen-specific TCRs. In principle, T cells expressing high affinity neoantigen-specific TCRs should be identifiable in the naïve T cell repertoire.

In this study, we aimed to identify MHC class I neoepitopes derived from mCALR and isolate TCRs against such neoepitopes with the potential to be utilized clinically for TCR gene therapy. For the purpose of epitope discovery we utilized a number of complimentary approaches. Firstly, we used MHC peptide elution mass spectrometry to try to identify potential mCALR neoepitopes. Secondly, we used a MHC class I binding peptide prediction algorithm to identify potential mCALR neoepitopes presented by common European Caucasoid HLA class I alleles, generated pMHC multimers encoding these predicted CALR peptides, and subsequently used combinatorial-encoded mCALR multimer staining to screen mCALR^+^ MPN patients for CD8^+^ mCALR T cell responses. We extended the MHC class I binding prediction analysis to include, in addition to *CALR*, some other mutations found recurrently in hematological malignancies e.g. *FBXW7*, *TP53*, and *MYD88*. These putative neoepitopes were used to generate a panel of pMHC multimers, to enable the isolation of neoepitope-specific CD8^+^ T cells from the T cell pool of readily available healthy human donor PBMC. Isolated putative neoepitope-specific T cells or TCRs were then analyzed for their ability to recognize cognate antigen in vitro.

## Methods

### Recurrent neoepitope peptide prediction

Putative mutated neoepitopes of 9, 10, or 11 amino acids in length were determined using NetMHC 3.4 (http://www.cbs.dtu.dk/services/NetMHC/). Neoepitopes were chosen based on their predicted ability to bind common European Caucasian HLA class I alleles (< 500 nM).

### MHC peptide elution mass spectrometry

Two healthy donor-derived Epstein-Barr virus (EBV)-transformed lymphoblastoid cell lines (LCLs), expressing a number of common HLA class I alleles (LCL 1 = HLA-A*01:01, −A*03:01, -B*08:01, -B*35:01 and LCL 2 = HLA-A*02:01, −A*11:01, -B*07:02, -B*44:02), were retrovirally transduced with a pMX-mCALR-IRES-eGFP or pMX-eGFP alone vector. GFP+ LCLs were sorted to a high purity and expanded to approximately 5 × 10^8^ cells before being washed with PBS and snap-frozen. Subsequent immunoprecipitation of HLA class I molecules, elution of bound peptides, and analysis of peptides by liquid chromatography tandem mass spectrometry (LC-MS/MS) was carried out as previously described [11]. Briefly, cell pellets were ground using a Retsch mixer mill (MM400) and then lysed in lysis buffer (0.5% IGEPAL CA-630 [Sigma-Aldrich], 50 mM Tris-HCl [pH 8.0], 150 mM NaCl, and protease inhibitors [complete protease inhibitor cocktail; Roche Life Science]) for 1 h at 4 °C. Lysate was pre-cleared by passing over unconjugated protein A sepharose [GE Healthcare] and then MHC:peptide complexes were captured by protein A-coupled pan-MHC class I-specific antibody W6/32. Dissociation of heavy chain, beta-2 microglobulin and peptide was then achieved using 10% acetic acid. Eluted MHC-peptides were subsequently purified by fractionation using reversed phased HPLC (RP-HPLC; Chromolith Speed Rod (Merck)) using an ÄKTAmicro HPLC system [GE Healthcare], as described elsewhere [11].

Fractionated peptides were vacuum concentrated using a Labconco Centrivac concentrator and resuspended to a final volume of 20 μL in 0.1% formic acid in water prior to LC-MS/MS analysis using an Eksigent NanoUltra cHiPLC system [SCIEX] (microfluidic trap column (200 μm × 0.5 mm ChromXP C18-CL [3 μm, 120 Å]) at a flow rate of 5 μL/min; microfluidic analytical column (75 μm × 15 cm ChromXP C18-CL [3 μm, 120 Å]) at a flow rate of 300 nL/min across a gradient of increasing concentration of acetonitrile in water supplemented with 0.1% formic acid) coupled to a TripleTOF® 5600+ mass spectrometer [SCIEX). Each scan cycle consisted of MS1 spectra acquired for 200 msec and the top 20 precursor ions fragmented using rolling collision energy and acquired for 150 msec. Mass spectra were searched using the Paragon™ algorithm of ProteinPilot™ v5 software [SCIEX]. A custom database was assembled using the combined Uniprot reference proteomes of *Homo sapiens* (v2016_06), enhanced green fluorescent protein, and mCALR sequences. Through the use of a decoy database, data were subjected to a 5% false discovery rate (FDR) cut-off.

### Neoepitope-specific T cell enrichment

Putative neoepitope HLA class I multimers were generated using conditional MHC class I ligands and peptide exchange technology [12]. Healthy donor PBMCs isolated from anonymised National Blood Service apheresis cones were incubated with phycoerythrin (PE)-labelled neoepitope multimers, before enrichment by magnetic-activated cell sorting (MACS) using anti-PE microbeads [Miltenyi Biotec]. Enriched cells were expanded using anti-CD3 mAb (30 ng/ml) [ebioscience] and IL-2 (3000 U/ml) [Novartis]. Neoepitope multimer^+^ CD8^+^ T cell populations were identified by combinatorial-encoded multimer staining [13]. To establish T cell clones, neoepitope multimer^+^ CD8^+^ cells were single-cell sorted and expanded using anti-CD3 mAb (30 ng/ml) [ebioscience] and IL-2 (3000 U/ml) [Novartis]. T cell clones were confirmed by neoepitope multimer and anti-CD8-APC (1:50) [BD] staining.

### Combinatorial-encoded multimer staining

For each putative mutated neoepitope being investigated, HLA class I multimers were labelled with a unique combination of two different streptavidin conjugated fluorochromes, to enable the simultaneous detection of two-colour combinations of multiple mutated neoepitope multimers in a single sample [13]. The streptavidin conjugates used were PE [BD], BB515 [BD], BV421 [BD], and BV650 [BD], allowing for 6 different two-colour combinations. For staining, either 3 μl of BV421 or PECy7, 2 μl of APC, BB515 OR BV650, or 1 μl of PE multimers per sample were taken into low-protein binding microcentrifuge tubes [Thermo Fisher Scientific] and centrifuged at 9600 x g for 2 min at 4 °C. The supernatants were transferred to fresh low-protein binding microcentrifuge tubes and adjusted to a volume of 50 μl with 5% HS, PBS. PBMCs were multimer stained as normal (Additional file 1), and also stained with anti-CD8-APC (1:50) [BD] and live/dead dye (1:200) [Life Technologies] for 30 min at 4 °C. PBMCs were then washed twice in 5% HS, PBS and resuspended in 200 μl of buffer for acquisition using an LSRII [BD] and analysis using FACSDiva software [BD].

### MPN patient mCALR multimer staining

MPN patient peripheral blood samples were obtained from the Queen Elizabeth Hospital Birmingham Clinical Haematology department. PBMCs were isolated from 30-40mls of MPN patient blood by density centrifugation over Ficoll-Paque [GE Healthcare]. Patient PBMCs were analyzed for HLA-A*03:01 and HLA-B*07:02 expression by PCR, as described in [14]. PBMCs from EBV^+^ healthy donors were also isolated and used for positive control EBV viral multimer staining. Putative mCALR peptides were used to generate HLA-A*03:01 and HLA-B*07:02 multimers. Patient PBMCs were analyzed for mCALR T cell responses by combinatorial-encoded multimer staining [13]. This study was conducted with ethical approval by the South Birmingham Local Research Ethics Committee and in accordance with the Helsinki Declaration.

### T cell reactivity assay

T cell reactivity was assessed by co-culturing T cells overnight with relevant peptide-loaded targets cells or cell lines, and detecting the T cell activation markers IFN-γ and CD137. LCLs were incubated with relevant or irrelevant peptides at a range of concentrations for 90 min at 37 °C, before being washed twice in excess media. Target cells and T cells were incubated overnight at a 1:1 ratio in triplicate cultures. Phorbol 12-myristate 13-acetate (PMA) (5 ng/ml) [Sigma-Aldrich] and ionomycin (500 ng/ml) [Sigma-Aldrich], or anti-CD3/CD28 human T cell activator dynabeads [Life Technologies], were used as a positive control. T cells cultured alone were used as a negative control. For intracellular IFN-γ staining, golgi plug [BD] was added (1 μl/ml). T cells were analyzed for IFN-γ and CD137 expression by flow cytometry the following day (Additional file 1).

### mCALR TCR retroviral plasmids

DNA was extracted from mCALR T cell clones using a DNeasy blood and tissue kit [Qiagen]. mCALR TCRs were sequenced using a high-throughput TCR gene capture technique [15]. Briefly, gDNA was sheared into 500 bp fragments and enriched for TCR genes by incubating with an RNA-bait library encoding baits against all TCR α and β variable gene loci. The enriched DNA fragments were then sequenced by paired-end (75–100 bp) Illumina sequencing. The output sequences were then aligned to a reference genome in order to identify TCR V(D)J genetic rearrangements and CDR3 sequences. The TCR α and β variable sequences were codon optimised for expression in human cells and synthesised and inserted into a retroviral pMP71 TCR flex vector by Genscript [15].

### Retroviral transduction

Healthy donor PBMCs or cell lines were transduced with retroviral plasmids by spinfection. Briefly, Phoenix Ampho cells were transfected with plasmid DNA and pcl ampho DNA using Fugene 6 transfection reagent [Promega]. Retrovirus was collected 48 h later, added to retronectin-coated plates [Takara], and centrifuged at 2000 g, 32 °C for 2 h. Activated PBMCs or cell lines were added to retrovirus-coated plates in appropriate media, centrifuged at 500 g for 5 min and incubated at 37 °C, 5% CO_2_. mCALR TCR transduction efficiency was determined by anti-mouse TCRβ-PE [1, 16] [Biolegend] and/or relevant dual-colour multimer staining. Cell line transduction efficiency was determined by GFP expression.

## Results

### MHC class I mCALR neoepitope discovery

Since recurrent *CALR* frameshift mutations result in the expression of a novel 36 amino acid C-terminus, we investigated whether this mutant region of the protein generates neoepitopes that are naturally processed and presented by common European Caucasoid MHC class I alleles. We used MHC class I peptide elution and mass spectrometry to identify neoepitopes expressed on the surface of LCLs retrovirally engineered with an eGFP tagged mCALR minigene, encoding the novel 36 amino acid sequence. These LCLs expressed a range of HLA class I alleles, including HLA-A*01:01, −A*03:01, −A*02:01, −A*11:01, -B*08:01, -B*35:01, -B*07:02 and -B*44:02. Following MHC isolation, peptide elution, and mass spectrometry analysis, we were able to identify several peptides derived from wildtype CALR and eGFP, however, no peptides derived from the 36 amino acid mCALR C terminus were detected (Additional file 2). Therefore, this data provides no evidence for the processing and presentation of mCALR peptides in the context of the HLA class I alleles studied.

### Identification of mCALR CD8^+^ T cell responses in MPN patients

**Fig. 1:**
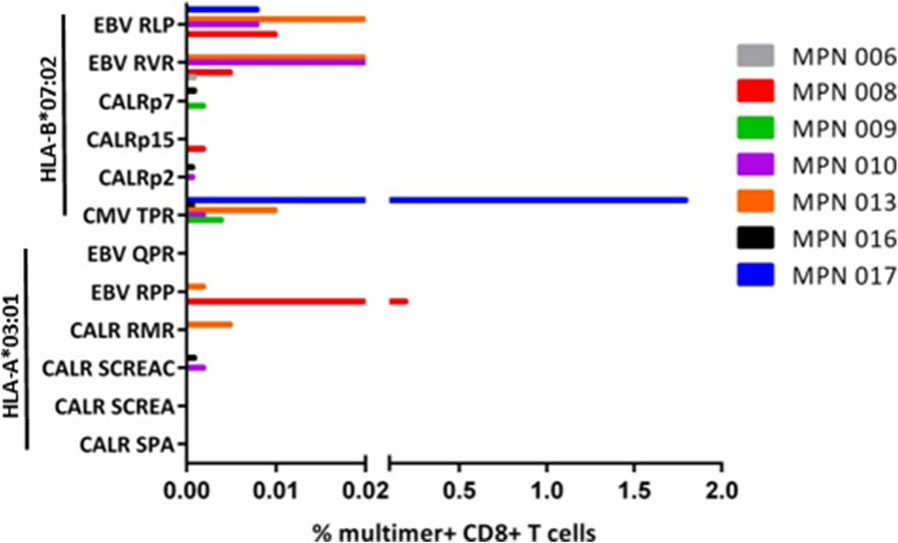
Investigation of putative mCALR CD8^+^ T cell populations in MPN. Combinatorial-encoded multimer staining was used to screen mCALR^+^ MPN patients for putative mCALR multimer^+^ CD8^+^ T cell populations in peripheral blood. Known cytomegalovirus (CMV)- and Epstein–Barr virus (EBV)- specific multimers were included as positive controls. Quantification of multimer^+^ CD8^+^ T cells within MPN patient PBMCs is shown

After failing to detect any mCALR MHC class I neoepitopes by mass spectrometry, we utilized the MHC peptide prediction algorithm NetMHC to identify putative mCALR MHC class I neoepitopes. A number of mCALR neoepitopes were predicted to bind strongly to HLA-A*03:01 and HLA-B*07:02 (Table 1). We then utilized MHC class I multimers containing these putative mCALR neoepitopes for multiplexed screening of mCALR T cell responses in the peripheral blood of HLA-A*03:01^+^ and/or HLA-B*07:02^+^ mCALR^+^ MPN patients (Additional file 3). While we could readily detect virus-specific multimer^+^ CD8^+^ T cell populations in PBMCs from 5 of the 7 MPN patients, putative mCALR multimer^+^ CD8^+^ T cell populations were undetectable in all patients, suggesting that these putative mCALR multimer^+^ CD8^+^ T cell populations are not, or very weakly, immunogenic (Fig. 1). It has been shown that T cell responses to naturally processed and presented antigens may not always be detectable within cancer patients [17–19]. Therefore, we began to explore if we could use a readily available source of material – healthy human donor material – to simultaneously identify mCALR neoepitopes and isolate TCRs targeting these neoepitopes.

**Table 1:** Putative HLA class I mCALR neoepitopes

Neoepitope	Peptide	Predicted HLA Restriction	Predicted HLA binding affinity (nM)
RPRTSCREA	CALR-REA	HLA-B*07:02	12
SPARPRTSC	CALR-SPA	HLA-B*07:02	22
RMRRTRRKM	CALR-RMR	HLA-B*07:02	56
RPRTSCREAC	CALR-REAC	HLA-B*07:02	23
RMMRTKMRMR	CALRp2	HLA-A*03:01	41
KMRMRRMRR	CALRp7	HLA-A*03:01	35
RTRRKMRRK	CALRp15	HLA-A*03:01	54

NetMHC was used to identify neoepitopes derived from the 36 amino acid mCALR C terminus that are predicted to bind strongly (< 500 nM) to common European Caucasoid HLA class I alleles

### Isolation and functional testing of mCALR-specific TCRs from healthy donor blood

**Fig. 2:**
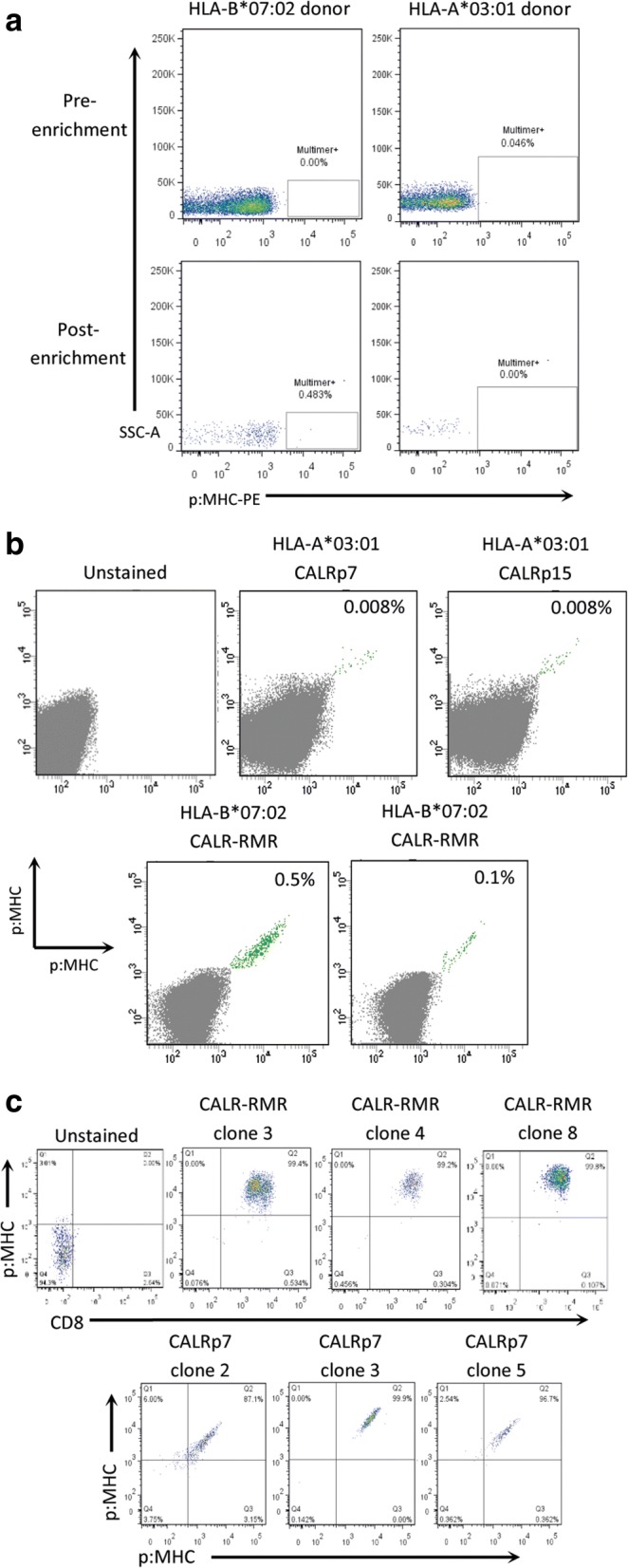
mCALR multimer^+^ CD8^+^ T cell isolation (**a**) Pools of HLA-A*03:01 and HLA-B*07:02 multimers containing putative mCALR neoepitopes were used to enrich CD8^+^ T cells from HLA-A*03:01^+^ and HLA-B*07:02^+^ healthy donor PBMCs. (*n* = 3) (**b**) Following enrichment and expansion of mCALR multimer^+^ T cells, numerous mCALR multimer^+^ CD8^+^ T cell populations were detected by combinatorial-encoded multimer staining. **c** mCALR multimer^+^ CD8^+^ T cell clones were isolated by single-cell sorting and identified by single or dual-colour multimer staining

We developed a protocol to isolate neoepitope-specific TCRs from PBMC of healthy human donors using MHC class I multimers (Additional file 4). We first demonstrated that this protocol could be used to successfully isolate very low frequency antigen-specific T cells by isolating HLA-A*02:01-restricted cancer-testes (CT) antigen-specific T cells from PBMC of HLA-A*02:01-expressing healthy human donors (Additional file 5). We then produced HLA-A*03:01 and HLA-B*07:02 PE-conjugated multimers containing the predicted mCALR neoepitopes to enrich for multimer-binding CD8^+^ T cells in HLA-A*03:01^+^ and HLA-B*07:02^+^ healthy donor PBMCs, respectively. A pool of putative neoepitope multimers were first used to enrich multimer-bound T cells from PBMC of healthy human donors (Fig. 2a). Directly following isolation we were unable to clearly detect enrichment of multimer-binding T cells in the small sample of cells taken for analysis, presumably because of the low precursor frequency of such T cells in the T cell pool of healthy human donors. Conversely, when a larger sample of cells were analyzed following expansion of multimer-enriched T cells, numerous mCALR multimer-binding CD8^+^ T cell populations could clearly be detected using combinatorial-encoded multimer staining (Fig. 2b). mCALR multimer^+^ CD8^+^ T cells were subsequently cloned by single-cell sorting and expanded using anti-CD3 antibodies and high dose IL-2 in a rapid expansion protocol. Utilizing this approach, we successfully isolated twenty CALR-RMR and three CALRp7 multimer^+^ CD8^+^ T cell clones which exhibited > 99% dual-colour multimer staining (Fig. 2c).

**Fig. 3:**
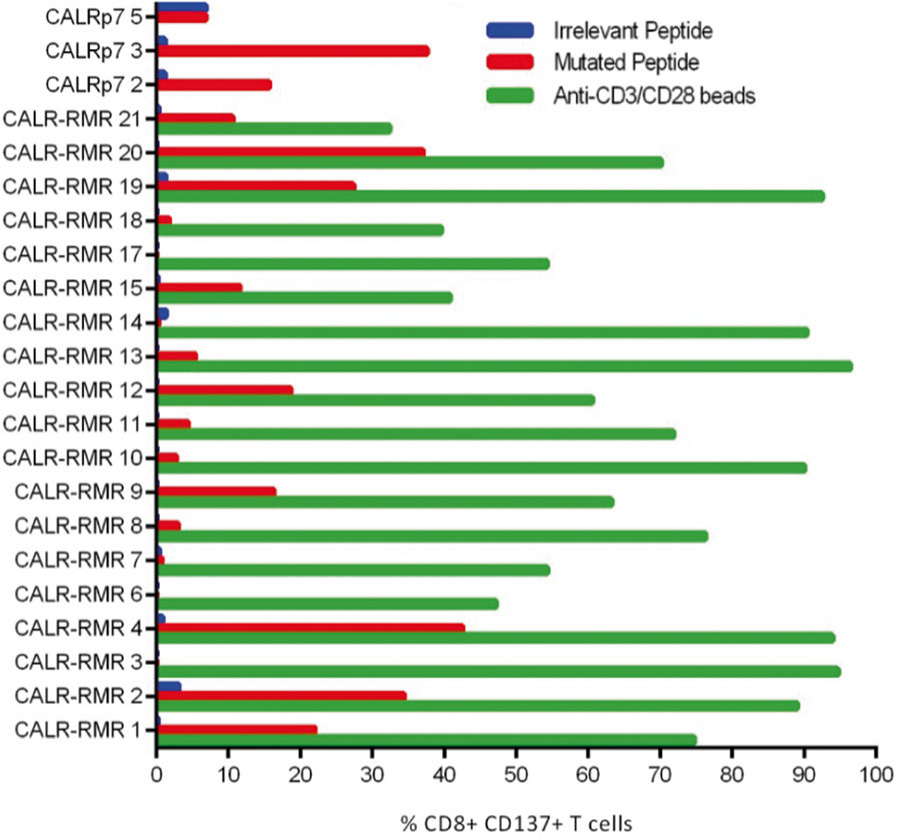
mCALR T cell clone peptide reactivity mCALR multimer^+^ CD8^+^ T cell clones were co-cultured with LCLs loaded with 10 μM of mCALR peptide or irrelevant peptide and assessed for reactivity by analysing CD137 expression by flow cytometry. CALR-RMR T cell clones were incubated with anti-CD3/CD28 beads as a positive control. (*n* = 1)

We initially assessed the functionality of mCALR multimer^+^ CD8^+^ T cell clones by assessing IFN-γ production and CD137 up-regulation following co-culture with lymphoblastoid cell lines (LCLs) loaded with relevant mCALR peptide (10 μM) or an irrelevant peptide (10 μM) (Fig. 3). The majority of T cell clones showed specific reactivity towards mCALR peptide (15/23), however a number of T cell clones did not, suggesting that the binding of multimer does not necessarily denote a functional T cell response, a phenomenon observed previously [20]. However, we successfully identified thirteen HLA-B*07:02-restricted CALR-RMR and two HLA-A*03:01-restriced CALRp7 reactive T cell clones. We proceeded to isolate the TCRαβ variable gene sequences from the highest IFN-γ-producing T cell clones (CALR-RMR clone 4, CALR-RMR clone 20, CALRp7 clone 2 and CALRp7 clone 3) using TCR gene capture technology (Additional file 6).

### mCALR TCR-engineering of healthy donor T cells

**Fig. 4:**
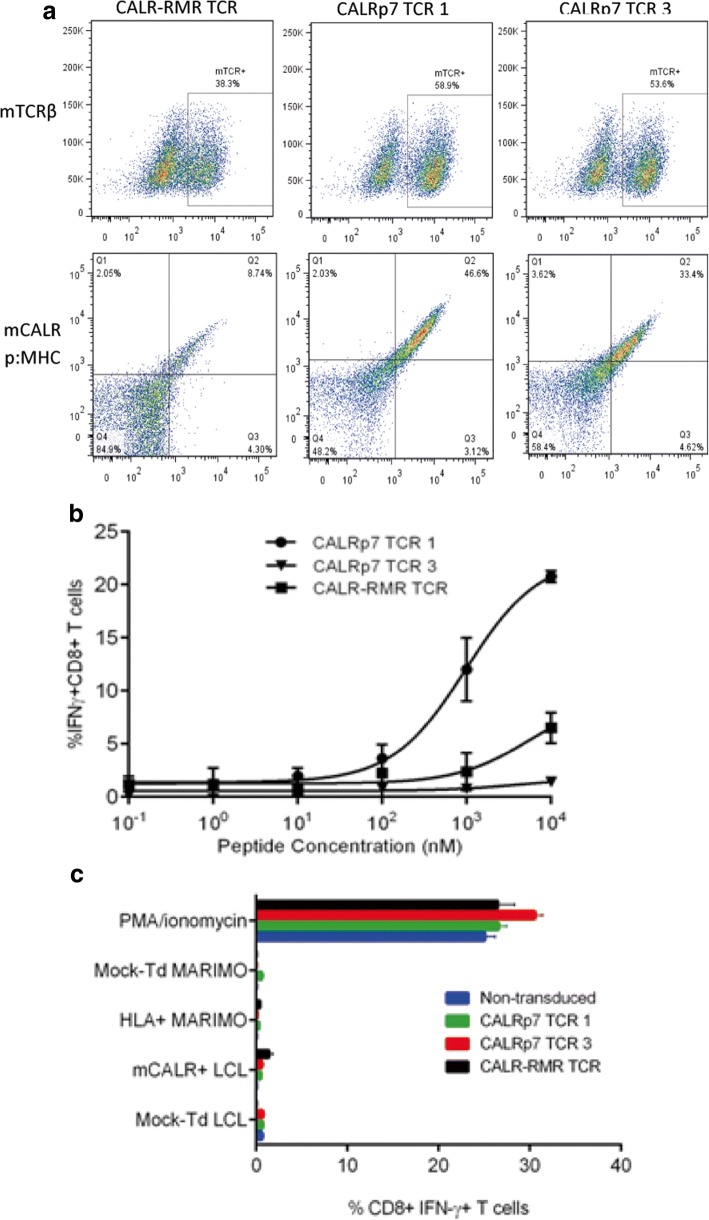
mCALR TCR-engineered T cell recognition of mCALR-expressing target cells (**a**) mCALR TCR transduction efficiency was determined by mouse TCRβ (mTCRβ) staining and dual-colour mCALR multimer staining. Mock-transduced T cells were used to determine positive staining. (*n* = 3) (**b**) mCALR TCR-engineered T cells were co-cultured with LCLs loaded with decreasing concentrations of mCALR peptide at a 1:1 ratio and assessed for reactivity by IFN-γ intracellular cytokine staining. (*n* = 3) (c) mCALR TCR-transduced T cells were co-cultured with mCALR-expressing target cells at a 1:1 ratio and assessed for reactivity by IFN-γ intracellular cytokine staining. (*n* = 3)

TCR sequencing revealed that the same TCR clonotype was identified in both of the sequenced CALR-RMR specific T cell clones (clone 4 and clone 20), which has been denoted CALR-RMR TCR. Three different TCR clonotypes were identified in the two sequenced CALRp7 T cell clones (clone 1 and clone 3), due to the expression of two different TCRα chains in CALRp7 T cell clone 3, denoted CALRp7 TCR 1, 2 and 3 (Additional file 6). Following CALRp7 multimer staining, only CALRp7 TCRs 1 and 3 showed specific binding (data not shown), therefore, only these CALRp7 TCRs were investigated further. The three pairs of isolated TCR α and β genes were each cloned into a pMP71 retroviral vector in a TCRβ-P2A-TCRα configuration utilizing fully murine TCR constant domains carrying an additional disulfide-bridge. The expression cassette was codon-optimized for expression in human cells. Healthy donor T cells were engineered with our mCALR TCRs using retroviral transduction. mCALR TCR surface expression was confirmed by mouse TCRβ staining and specific mCALR multimer staining (Fig. 4a). Transduction efficiencies ranged from 58.9–38.3% using mouse TCRβ staining, and 46.6–8.74% using multimer staining. Of note, the percentage of multimer^+^ CD8^+^ T cells was significantly lower than the percentage of mouse TCRβ^+^ CD8^+^ T cells for CALR-RMR TCR, suggesting the CALR-RMR TCR may have a particularly low affinity for CALR-RMR multimer binding. We assessed the antigen sensitivity of T cells engineered with mCALR TCRs by co-culture with LCLs loaded with decreasing concentrations of mCALR peptide and measured IFN-γ production by intracellular staining (Fig. 4b). CALRp7 TCR 3 showed no reactivity against CALRp7 peptide at any concentration tested. In contrast, CALR-RMR and CALRp7 TCR 1 displayed peptide reactivity with EC50 values of 5487 nM and 975 nM, respectively. However, it should be noted that these EC50 values may be underestimated, as it is not clear if these T cell responses are maximal at 10^4^nM. These data suggest that both of these TCRs display a low affinity for their respective p:MHC complexes. To determine if the mCALR TCRs were able to recognize processed and presented mCALR antigen, we engineered HLA-A*03:01^+^ HLA-B*07:02^+^ LCLs with an eGFP tagged mCALR minigene, which was confirmed by RT-PCR (Additional file 7). Additionally, we engineered MARIMO cells, an essential thrombocythaemia cell line known to express a type 1 CALR frameshift mutation (c.1099_1159del; L367 fs*43), with HLA-A*03:01- and HLA-B*07:02-expressing retroviral vectors (Additional file 7). However, mCALR TCR-engineered T cells were unable to recognize mCALR-expressing target cells (Fig. 4c). The lack of recognition of the mCALR expressing cell lines by the TCR engineered T cells could have been due to their low affinity for mCALR peptide:MHC, or alternatively may be due to a lack of processing and presentation of these putative mCALR peptides in the context of HLA-A*03:01 and HLA-B*07:02.

### Isolation of putative neoepitope TCRs targeting recurrent mutations in hematological malignancies

**Fig. 5:**
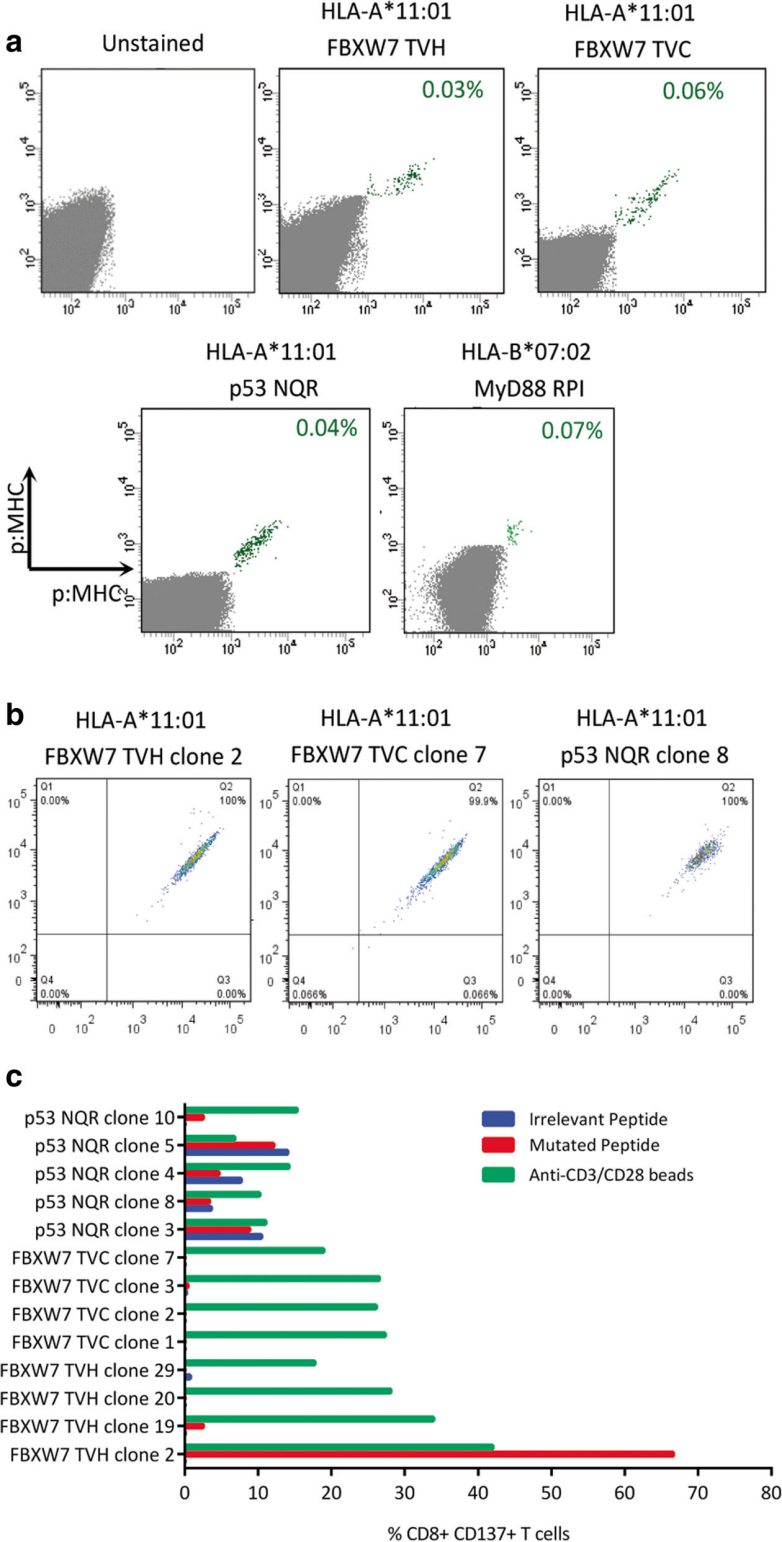
Isolation of additional putative neoepitope TCRs targeting recurrent mutations in hematological malignancies (**a**) Neoepitope-specific CD8^+^ T cells were enriched using neoepitope-bound multimers. After expansion of enriched cells, neoepitope multimer^+^ CD8^+^ T cell populations could be detected by combinatorial-encoded multimer staining. (*n* = 1) (**b**) Neoepitope multimer^+^ CD8^+^ T cells were cloned by single-cell sorting. Four FBXW7 TVH, four FBXW7 TVC and five p53 NQR CD8^+^ T cell clones were isolated. Representative multimer staining flow plots are shown with dual-colour multimer staining shown on the x and y axis. **c** Neoepitope T cell clones were assessed for reactivity against neoepitope peptide- or irrelevant peptide- loaded LCLs (10 μM) by CD137 staining. (*n* = 1)

We next turned our attention to other mutations found recurrently in hematological malignancies. We firstly identified somatic mutations found recurrently in hematological malignancies using the catalogue of somatic mutations in cancer (COSMIC). As before, we utilized the MHC peptide prediction algorithm NetMHC to identify putative neoepitopes encoding these mutations that were predicted to bind strongly to common European Caucasoid HLA class I alleles. We found a number of neoepitopes predicted to bind to HLA-A*11:01 and HLA-B*07:02 (Table 2). These included neoepitopes derived from hot-spot mutations in the tumour suppressor genes *FBXW7* and *TP53*, and also point mutations in *MYD88*, *IDH2*, *DNMT3A* and *STAT3*. We then generated multimers containing these neoepitopes to enrich for neoepitope-specific T cells from healthy donor PBMC. Following enrichment and expansion, we detected FBXW7 TVH, FBXW7 TVC, TP53 NQR and MYD88 RPI multimer^+^ CD8^+^ T cell populations using combinatorial-encoded multimer staining (Fig. 5a). The cloning of these multimer^+^ CD8^+^ T cell populations resulted in the isolation of four FBXW7 TVH-, four FBXW7 TVC-, and five p53 NQR-specific CD8^+^ T cell clones, which exhibited bright dual-colour multimer staining (Fig. 5b). We then assessed the reactivity of these T cell clones against peptide-loaded LCLs using IFN-γ intracellular cytokine staining, and found that only one T cell clone displayed specific peptide reactivity, namely FBXW7 TVH clone 2, as determined by CD137 upregulation upon stimulation (Fig. 5c).

**Table 2:** Putative neoepitopes encoding mutations found recurrently in hematological cancers

Protein	Mutation	Putative epitope	Peptide	HLA-Restriction	Predicted HLA binding affinity (nM)	Hematopoietic and Lymphoid Cancer Frequencies
CALR	K385Nfs*47	RPRTSCREA	CALR-REA	HLA-B*07:02	12	Essential thrombocythemia (31.1%), Myelofibrosis (27.3%)
		SPARPRTSC	CALR-SPA	HLA-B*07:02	22	
		RMRRTRRKM	CALR-RMR	HLA-B*07:02	56	
		RPRTSCREAC	CALR-REAC	HLA-B*07:02	23	
		RMMRTKMRMR	CALRp2	HLA-A*03:01	41	
		KMRMRRMRR	CALRp7	HLA-A*03:01	35	
		RTRRKMRRK	CALRp15	HLA-A*03:01	54	
FBXW7	R465C	TVCCMHLHEK	TVC	HLA-A*11:01	29	T-ALL (15.4%), Precursor T cell lymphoblastic lymphoma (15.6%)
		STVCCMHLHEK	STV	HLA-A*11:01	65	
		HTSTVCCMHLH	HTS	HLA-A*11:01	495	
	R465H	STVHCMHLH	STVH	HLA-A*11:01	78	
		TVHCMHLHEK	TVH	HLA-A*11:01	43	
P53	R248Q	SSCMGGMNQR	NQR	HLA-A*11:01	177	Mantle cell lymphoma (9.1%), B-ALL (6.9%), T-ALL (8.6%), Follicular lymphoma (18.5%), AML (7%), MDS (7.3%), CLL (10.9%), T cell lymphoma (18.6%), Burkitt’s lymphoma (18%), Diffuse large B cell lymphoma (12.6%)
	R248W	SSCMGGMNWR	NWR	HLA-A*11:01	320	
MyD88	L265P	RPIPIKYKAM	RPI	HLA-B*07:02	20	MGUS (46.8%), Waldenström’s macroglobulinaemia (86.3%), Diffuse large B cell lymphoma (14.4%)
		SPGAHQKRPI	SPG	HLA-B*07:02	40	
IDH2	R140Q	SPNGTIQNIL	SPN	HLA-B*07:02	72	AML (9.7%), Angioimmunoblastic T cell lymphoma (24.1%)
DNMT3A	R882H	VSNMSHLAR	VSN	HLA-A*11:01	61	AML (20.4%), MDS (9.1%), T cell lymphoma (25.7%),
STAT3	Y640F	QIQSVEPFTK	QIQ	HLA-A*11:01	60	T cell large granular lymphocytic leukaemia (34.3%), Adult T-cell leukemia/lymphoma (21.1%)

The catalogue of somatic mutations in cancer (COSMIC) was used to identify mutations found recurrently in hematological cancers. The MHC binding algorithm NetMHC3.4 was utilized to identify putative neoepitopes encoding these mutations that were predicted to bind strongly to common European Caucasoid HLA class I alleles. *T-ALL* T cell acute lymphoblastic leukemia, *B-ALL* B cell acute lymphoblastic leukemia, *AML* acute myeloid leukemia, *MDS* myelodysplastic syndrome, *CLL* chronic lymphocytic leukemia, *MGUS* monoclonal gammopathy of undetermined significance

### Functional testing of mutant FBXW7-specific TCRs from healthy donor blood

**Fig. 6:**
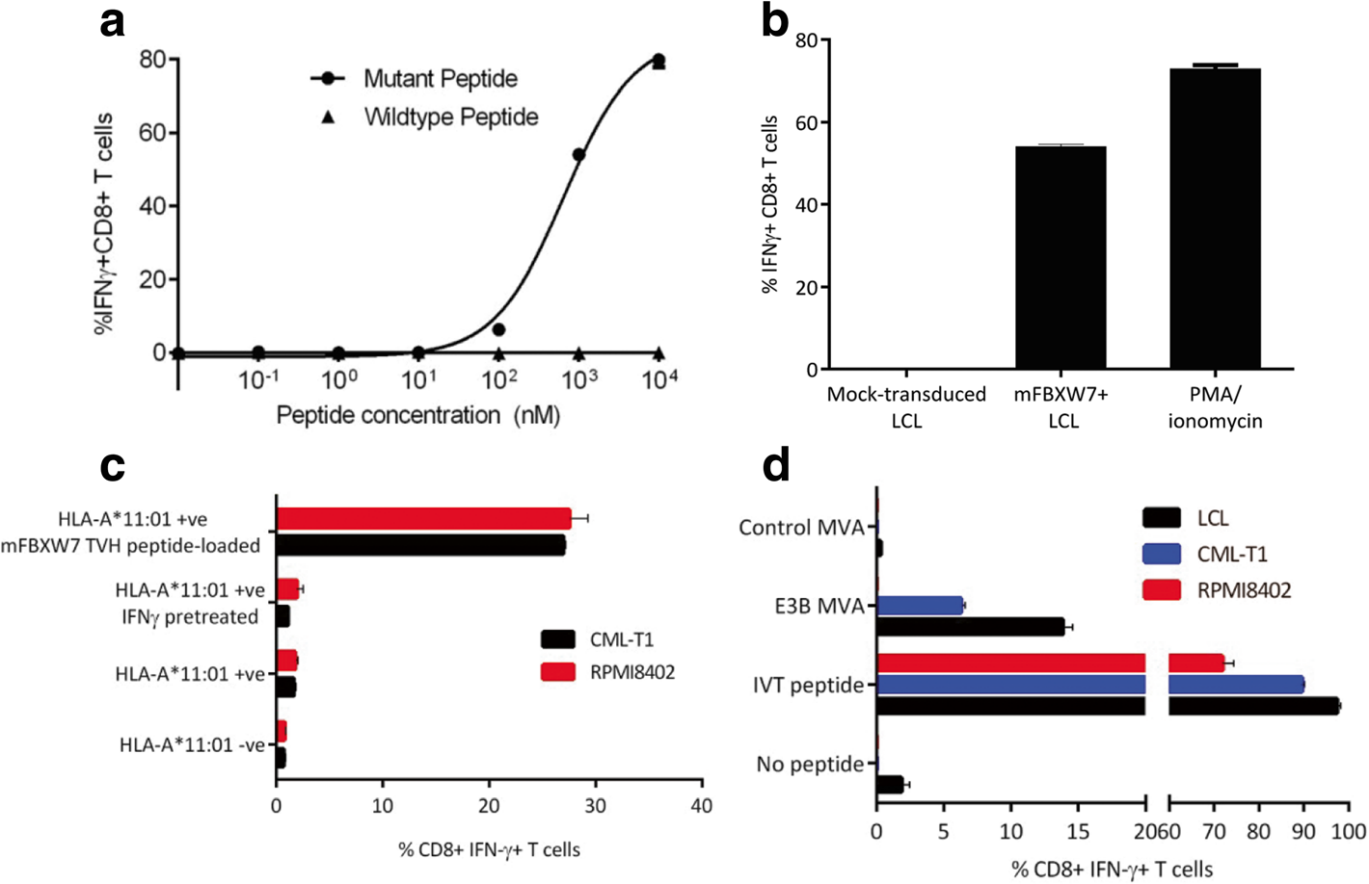
Mutant FBXW7 TVH clone 2 recognizes naturally processed and presented mFBXW7 antigen (**a**) FBXW7 TVH clone 2 was co-cultured with LCLs loaded with decreasing concentrations of FBXW7 mutant or wildtype peptide at a 1:1 ratio. Reactivity was determined by IFN-γ intracellular cytokine staining. (*n* = 3) (**b**) FBXW7 TVH clone 2 was co-cultured with mFBXW7^+^ LCL or mock-transduced LCL at a 1:1 ratio. Reactivity was determined by IFN-γ intracellular cytokine staining. (*n* = 3) (**c**) FBXW7 TVH clone 2 was co-cultured with HLA-A*11:01^+^ CML-T1 or HLA-A*11:01^+^ RPMI8402, with or without IFN-γ pre-treatment, at a 1:1 ratio. Reactivity was determined by IFN-γ intracellular cytokine staining. (*n* = 2) (**d**) HLA-A*11:01^+^ CML-T1, HLA-A*11:01^+^ RPMI8402 or HLA-A*11:01^+^ LCLs were infected with an EBNA3B MVA encoding the EBV derived epitope IVT, or an empty control MVA. IVT-specific CD8^+^ T cells were co-cultured with EBNA3B MVA infected target cells at a 1:1 ratio. Reactivity was determined by IFN-γ intracellular cytokine staining. (*n* = 2)

Encouraged by the response of TVH clone 2 against the mutated peptide, we next determined whether FBXW7 TVH clone 2 was able to discriminate between the mutant FBXW7 (mFBXW7) TVH peptide and the wildtype FBXW7 peptide, in which the histidine residue at P3 was reverted back to the wildtype arginine residue. For this purpose, T cells were co-cultured with LCLs loaded with decreasing concentrations of either the mutated or wildtype FBXW7 peptide and IFN-γ production was assessed by intracellular cytokine staining. FBXW7 TVH clone 2 showed no recognition of LCLs loaded with any concentration of wildtype peptide, however, it displayed specific recognition of LCLs loaded with mFBXW7 TVH peptide (EC50 value 648 nM) (Fig. 6a). These results demonstrate that the TCR expressed by this T cell clone can discriminate between the mutant and WT peptides, displaying fine peptide specificity towards the mFBXW7 peptide with a single amino acid substitution.

We then sought to determine if the putative mFBXW7 TVH neoepitope recognized by FBXW7 TVH clone 2 could be processed and presented in the context of HLA-A*11:01. For this purpose we engineered HLA-A*11:01^+^ LCLs with an eGFP tagged mFBXW7 minigene encoding the c.1394 G > A p.R465H mutation flanked by 15 wildtype nucleotides (mFBXW7^+^ LCL), and confirmed mFBXW7 expression by RT-PCR (Additional file 8). Encouragingly, following co-culture, FBXW7 TVH clone 2 showed reactivity towards mFBXW7^+^ LCLs, but showed no reactivity towards non-engineered LCL expressing only wildtype FBXW7 (Fig. 6b). These findings suggest that the FBXW7 TVH neoepitope encoding the R465H mutation is capable of being processed and presented on the surface of target cells by HLA-A*11:01, for recognition by FBXW7 clone 2 T cells.

Finally, we assessed whether FBXW7 TVH clone 2 can recognize tumour cells naturally expressing endogenous levels of FBXW7 R465H mutated protein. Therefore, we engineered two T cell acute lymphoblastic leukaemia (T-ALL) cell lines (CML-T1 and RPMI8402), with confirmed FBXW7 c.1394 G > A p.R465H mutant protein expression, with an HLA-A*11:01 retroviral vector (Additional file 8). Prior to T cell-tumor cell co-culture, some tumor cells were pre-treated with 200 IU/ml rh-IFN-γ for 24 h, as this was shown to induce upregulation of MHC (data not shown), indicative of upregulation of the immunoproteasome. Following co-culture, FBXW7 TVH clone 2 did not show reactivity towards T-ALL cell lines, whether untreated or pre-treated with IFN-*γ* (Fig. 6c).

The lack of recognition of T-ALL cell lines may potentially be due to defects in antigen processing and presentation. As FBXW7 provides substrate recognition to a multi-subunit ubiquitin ligase complex that is responsible for ubiquitin-dependent degradation of proteins, mutations in FBXW7 may adversely affect proteasomal processing and presentation of antigens, including FBXW7 itself. Therefore, to determine whether CML-T1 and RPMI8402 cells have any global antigen presenting defects, we infected cells with an EBNA3B modified vaccinia ankara (MVA) virus encoding the EBV nuclear antigen 3B (EBNA3B), which contains the IVT epitope (EBNA3B416–424) restricted through HLA-A*11:01 [21]. The infected cells were then co-cultured with an EBNA3B IVT-reactive T cell clone (Fig. 6d). We found that IVT-reactive T cells were able to recognize CML-T1 EBNA3B MVA infected cells, but not RPMI8402 EBNA3B MVA infected cells, suggesting that RPMI8402 cells, but not CML-T1 cells, are defective in processing antigen. Therefore, this may explain the lack of recognition of RPMI8402 cells by FBXW7 TVH clone 2, but does not explain their lack of recognition of CML-T1. Furthermore, the lack of recognition of the CML-T1 and RPMI8402 may be due to a relatively low affinity for FBXW7 TVH peptide, thereby preventing it from recognizing physiological levels of the FBXW7 TVH presented on tumor cells.

## Discussion

The purpose of this study was to simultaneously identify neoepitopes containing somatic cancer mutations found recurrently in hematological malignancies, and TCRs reactive against such neoepitopes from the healthy human donor T cell pool. mCALR was initially chosen as an attractive target for immunotherapy, given its expression of a novel stretch of 36 amino acids, and its high frequency in *JAK2wt* MPN patients (~ 80%) [7, 8]. However, our results failed to detect any mCALR neoepitopes presented by common European Caucasoid HLA class I alleles using MHC peptide elution mass spectrometry. Additionally, using MHC class I multimer staining against predicted mCALR epitopes, mCALR CD8^+^ T cells were undetectable in the small number of mCALR^+^ MPN patient PBMC we screened. Interestingly, CD8^+^ T cells with reactivity against putative mCALR neoepitopes could be repeatedly enriched from the healthy human donor T cell pool. There is currently no evidence to suggest that mCALR induces tolerance in mCALR^+^ patients, and of interest, our mCALR-derived neoepitopes are not shared with any self-peptides. It is most likely however, that following mCALR multimer enrichment and multiple rounds of stimulation, we enriched mCALR-specific T cells from healthy donor blood, where they were presumed to be of a naïve T cell precursor frequency. On the other hand, we performed mCALR multimer staining of mCALR^+^ patient PBMC directly ex vivo, without prior enrichment and expansion, where antigen-specific T cells of naïve frequency would be under the detection limit of multimers.

Although putative mCALR-specific TCRs showed reactivity towards cognate peptide, they were unable to recognize naturally processed and presented mCALR^+^ target cells. Taken together, we found no evidence that the predicted mCALR peptides studied are naturally processed and presented in the context of the HLA class I alleles investigated. However, since the mCALR C-terminus contains many hydrophilic residues, which are particularly difficult to bind and separate using C18 reverse-phase HPLC, a false-negative result is possible, and is a caveat of using this type of approach for epitope discovery.

Although this study focused on CD8^+^ T cells, CD4^+^ T cells, which are classically described as helper T cells, can also exhibit direct anti-tumour cytotoxic function in both mice and humans [22, 23]. Numerous studies have highlighted the contribution of tumour-reactive CD4^+^ T cells to cancer immunotherapy efficacy [1, 24, 25]. Interestingly, CD4^+^ T cell responses against long mCALR peptides that contain our minimal mCALR epitopes of interest (CALR-RMR and CALRp7) have recently been discovered in the blood of mCALR^+^ MPN patients [16]. However, when Holmström et al. performed mCALR minimal epitope screening of T cells reactive to long mCALR peptides by IFN-γ ELIspot, CALR-RMR and CALRp7 peptides were not included. In addition, an HLA-DR-restricted TCR was isolated which was able to recognize autologous mCALR^+^ hematopoietic stem cells and mCALR^+^ monocytes, suggesting that mCALR can give rise to specific TCRs with therapeutic potential for treating mCALR^+^ MPN patients [16, 26]. However, in agreement with our findings, the study by Holmström et al. was also unable to isolate CD8^+^ mCALR-reactive T cell clones. These findings suggest that mCALR is a promising target for CD4^+^ T cell based immunotherapy. However, whether mCALR is targeted by CD8^+^ T cells warrants further investigation. It is also important to note that CALR plays a key role in the MHC class I processing pathway, and thus mutations in CALR may interfere with this process. Hence, detection of MHC class II-restricted responses to mCALR, may be more likely than MHC class I responses.

After turning our attention to other mutations found recurrently in hematological malignancies, we successfully identified a CD8^+^ T cell clone with reactivity against the R465H hot-spot mutation in FBXW7. *FBXW7* is among one of the most frequently mutated cancer genes, with a cancer-wide average frequency of 6%, and a frequency of 15–31% in T-ALL [27]. FBXW7 is an F-box protein family member that provides substrate recognition to the SCF ubiquitin ligase complex. The SCF ubiquitin ligase complex is responsible for the ubiquitination of proteins destined for proteasomal degradation, and is therefore important in many cellular processes, including cellular division and cell cycle progression. *FBXW7* has been identified as a tumour-suppressor gene following studies showing its role in the ubiquitin-dependent degradation of a number of oncoproteins [28]. It is therefore unsurprising that loss-of-function mutations within *FBXW7* are found recurrently in a diverse range of cancers. CD8^+^ mFBXW7-specific T cells showed specific recognition of mutant peptide and engineered target cells, suggesting that the mFBXW7 peptide is naturally processed and presented, and suggesting the discovery of a novel neoepitope. However, the particular T cell clone isolated was unable to recognize mFBXW7^+^ T-ALL cell lines, possibly owing to the relatively low affinity of its TCR for mFBXW7 peptide:MHC complex. Another possibility is that mutations in FBXW7 may affect the proteasomal processing of the FBXW7 protein itself, preventing FBXW7 antigen processing and presentation. We attempted to address this possibility (Fig. 6d), where we found that CML-T1 cells, but not RPMI8402 cells, were able to present the EBV antigen EBNA3B and activate EBNA3B-specific T cells. Interestingly, CML-T1 cells were found to be heterozygous for the FBXW7 R465H mutation, whereas RPMI8402 cells are homozygous (data not shown). This finding may suggest that FBXW7 R465H causes deficiency in antigen processing and presentation, but that a single copy of wildtype FBXW7 is able to rescue this phenotype in CML-T1 cells. Further work is therefore needed to conclude whether FBXW7 R465H causes global antigen processing and presentation defects, and thus determine if mFBXW7 is a suitable target antigen for immunotherapy.

Throughout this study we utilized conditional MHC class I ligands and peptide exchange technology to allow the flexible and high-throughput generation of neoepitope-bound MHC class I multimers for enrichment of neoepitope-specific CD8^+^ T cells [12]. Using this method, we isolated numerous multimer^+^ CD8^+^ T cell clones, however, a significant number of these showed no reactivity against relevant peptide-loaded LCLs, demonstrating that multimer binding does not always indicate functional reactivity. However, it should be noted that the sole use of CD137 and IFN-γ for assessing T cell reactivity might have underestimated the number of neoepitope-reactive CD8^+^ T cell clones expressing alternative cytokines and/or activation markers. Although multimers are powerful tools for isolating antigen-specific T cells, we encountered two limitations in their use: 1. the isolation of low-affinity antigen-specific T cells, and 2. the peptide complexes in question may not be naturally processed in target cells. Therefore, whilst this approach successfully identified a novel neoepitope (mFBXW7 TVH), it is not well suited to simultaneous epitope and high-affinity TCR discovery, since the identification of high affinity TCRs is important for therapeutic efficacy.

An alternative approach would involve the induction of neoepitope-specific T cell responses using antigen-specific stimulation e.g. autologous antigen presenting cells transfected with neoepitope minigenes. An induction approach would select those TCRs with reactivity against neoantigens that have been naturally processed and presented, therefore likely reducing the number of low-affinity non-functional TCRs isolated, and increasing TCR discovery efficiency. This type of approach has been successfully used by others to isolate neoantigen-reactive T cells from the naïve T cell repertoires of healthy blood donors [17], therefore using this approach may uncover many more high affinity recurrent neoepitope-reactive TCRs with greater therapeutic potential.

## Conclusions

We investigated whether mutations found recurrently in hematological malignancies encode immunogenic neoantigens presented by common European Caucasoid HLA class I alleles. While mCALR did not appear to be immunogenic in the context of the HLA class I alleles investigated, we isolated an mFBXW7-specific TCR with reactivity against mFBXW7-engineered target cells, suggesting the identification of a novel neoepitope expressed recurrently in a diverse range of cancers, including T-ALL.

## Additional files

Additional file 1: Supplementary materials and methods. (DOCX 221 kb)

Additional file 2: Identification of peptides derived from wildtype CALR and eGFP but not mCALR. (DOCX 17 kb)

Additional file 3: Myeloproliferative neoplasm patient cohort. (DOCX 16 kb)

Additional file 4: Neoepitope-specific T cell enrichment work flow. (DOCX 233 kb)

Additional file 5: Detection of cancer-testes antigen T cell responses in the healthy donor T cell repertoire. (DOCX 1988 kb)

Additional file 6: mCALR-specific TCR gene rearrangments. (DOCX 1896 kb)

Additional file 7: Engineering of mCALR-expressing target cells. (DOCX 488 kb)

Additional file 8: Engineering of mFBXW7-expressing target cells. (DOCX 409 kb)

## Abbreviations

COSMIC: Catalogue of somatic mutations in cancer
CT: Cancer-testes
EBNA3B: EBV nuclear antigen 3B
EBV: Epstein-Barr virus
HPLC: High-performance liquid chromatography
LCL: Lymphoblastoid cell line
mCALR: Mutant CALR
mFBXW7: Mutant FBXW7
MHC: Major histocompatibility complexes (MHC)
MPN: Myeloproliferative neoplasm
MVA: Modified vaccinia Ankara
PE: Phycoerythrin
T-ALL: T cell acute lymphoblastic leukaemia
TCR: T cell receptor
