# Supplementary material for: Isolation of T cell receptors targeting recurrent neoantigens in hematological malignancies
Source: J Immunother Cancer. 2018 Jul 13;6:70. doi: 10.1186/s40425-018-0386-y (PMC6044029; doi:10.1186/s40425-018-0386-y)
Supplement: Supplementary file 1 — Supplementary materials and methods. (DOCX 221 kb) [file 40425_2018_386_MOESM1_ESM.docx]

Additional file 1

**Supplementary Methods**

**Multimer staining**

T cell populations were stained with either 1μl of PE-labelled multimers, 2μl of allophycocyanin (APC)-, BB515- or BV650-labelled multimers or 3μl of PeCy7- or BV421-labelled multimers, in 50μl of 5% HS, PBS, for 15 minutes at 37°C. Additional surface antibody staining was then performed as normal by exposing cells to pre-determined concentrations of antibody for 30 minutes at 4°C. T cells were washed twice with 5% HS, PBS, before being resuspended in 200μl of 5% HS, PBS, acquired on an LSRII [BD], and analyzed using FlowJo software [Treestar].

**IFN-γ intracellular cytokine staining**

T cells were washed in FACS buffer (5% FCS, PBS) and stained with anti-CD8-PE (1:50) [BD] and live/dead dye (1:200) [Life Technologies] for 30 minutes at 4°C. T cells were then washed in FACS buffer and resuspended in 100μl of Cytofix/Cytoperm Fixation/Permeabilization Solution [BD] for 20 minutes at 4°C. T cells were then washed twice in 1X Perm/Wash Buffer [BD] and stained intracellularly with anti-IFN-γ-APC (1:200) [Biolegend] in 1X Perm/Wash Buffer, for 30 minutes at 4°C. Cells were then washed twice in 1X Perm/Wash Buffer, and resuspended in 200μl of FACS buffer for acquisition on an LSRII [BD] and analysis using FlowJo software [Treestar].

**CD137 T cell staining**

Expression of the T cell activation marker CD137 following co-culture with target cells was assessed by flow cytometry. Cells were pelleted at 500 x g for 5 minutes and washed in FACS buffer (5% FCS, PBS). Cells were stained with anti-CD8-FITC (1:25) [Biolegend], live/dead dye (1:200) [Life Technologies] and anti-CD137-APC (1:25) [BD], for 30 minutes at 4°C. Cells were then washed twice with FACS buffer and resuspended in 200μl of FACS buffer. Cells were acquired on an LSRII [BD] and analyzed using FlowJo software [Treestar].

**Generation of mCALR and mFBXW7 Retroviral Plasmids**

mCALR and mFBXW7 gene fragments containing a 5’ BamHI restriction site and a 3’ NotI restriction site were codon optimised for expression in human cells and synthesised by Genscript. The mCALR-GFP retroviral plasmid was constructed by digesting a pMX-IRES-GFP plasmid (Cell Biolabs, CA, USA) with BamHI and NotI fast digest restriction enzymes [Thermo Fisher Scientific], according to manufacturers’ instructions. The digested pMX-IRES-GFP backbone was gel purified by running the digested DNA product on a 1% agarose gel, cutting out the appropriate size band and retrieving the DNA using the QIAquick Gel Extraction Kit [Qiagen]. The mCALR gene fragment was then ligated into the pMX-IRES-GFP backbone using DNA ligase [NEB], according to manufacturer’s instructions. The pMX-mFBXW7-IRES-GFP retroviral plasmid was constructed by Genscript in the interest of time. Following preparation of mCALR-GFP and mFBXW7-GFP retroviral plasmids by Maxiprep [Qiagen], plasmid DNA was sequenced using a pMX-specific forward primer (5’ GGCATCGCAGCTTGGATACACG 3’).

**HLA Retroviral Plasmids**

pmx-HLA-A*03:01-IRES-GFP, pmx-HLA-B*07:01-IRES-GFP, and pmx-HLA-A*11:01 retroviral plasmids were a kind gift from Ton Schumacher at the Netherlands Cancer Institute.

**Detecting mCALR and mFBXW7 RNA Transcripts by RT-PCR**


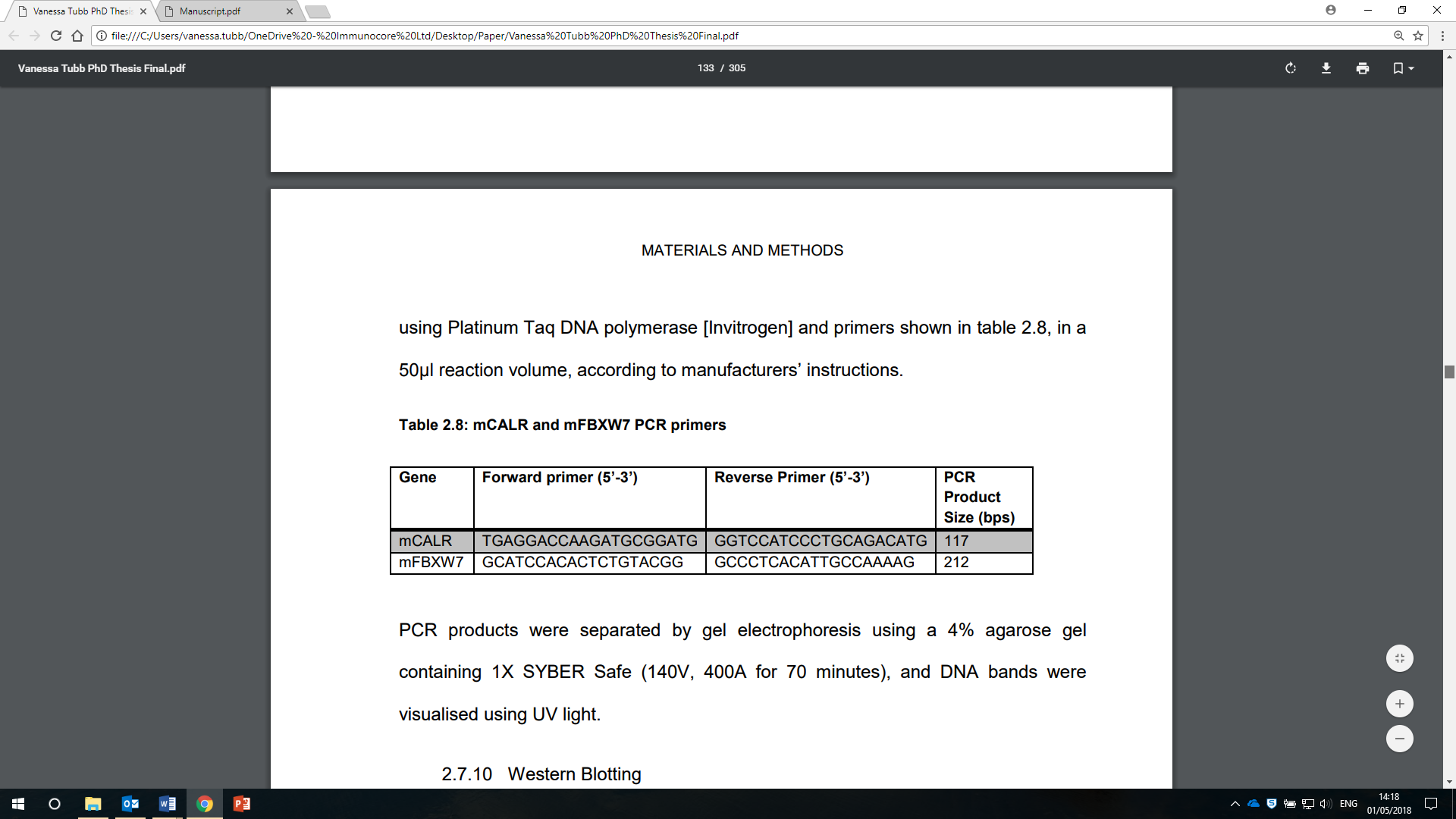
RNA was extracted from LCLs engineered to express mCALR and mFBXW7 retroviral plasmids, and treated with DNase [Ambion] according to the manufacturers’ instructions, to remove contaminating genomic DNA. RNA was then used as a template to generate cDNA using an oligo(dT)12-18 primer and the Superscript III Reverse Transcriptase kit [Invitrogen] according to manufacturers’ instructions. mCALR or mFBXW7 PCR was then performed using mCALR and mFBXW7 specific primers (see table below) and Platinum Taq DNA polymerase [Invitrogen] in a 50µl volume, according to manufacturers’ instructions. PCR products were separated by gel electrophoresis using a 4% agarose gel containing 1X SYBER Safe (140V, 400A for 70 minutes), and DNA bands were visualised using UV light.
