# Supplementary material for: Isolation of T cell receptors targeting recurrent neoantigens in hematological malignancies
Source: J Immunother Cancer. 2018 Jul 13;6:70. doi: 10.1186/s40425-018-0386-y (PMC6044029; doi:10.1186/s40425-018-0386-y)
Supplement: Supplementary file 2 — Identification of peptides derived from wildtype CALR and eGFP but not mCALR. (DOCX 17 kb) [file 40425_2018_386_MOESM2_ESM.docx]

Additional file 2

**Identification of GFP- and wildtype CALR-derived peptides, but not mCALR-derived peptides by MHC peptide elution mass spectrometry:** MHC class I-bound peptides were eluted from prepared mCALR-GFP expressing LCL samples by C18 reverse-phase high-performance liquid chromatography (HPLC), and analyzed by LC-MS/MS using an SCIEX TripleTOF(R) 5600+ system. A confidence ranking system was employed and a false discovery rate (FDR) cut-off of 5% was applied to the mass spectral data (LCL 1 GFP FDR = 96.2%, LCL 1 mCALR-GFP FDR = 95.9%, LCL 2 GFP FDR = 95.9% and LCL 2 mCALR-GFP FDR = 95.9%). Peptide sequences derived from wildtype calreticulin and enhanced green fluorescent protein could be detected, however, mCALR-derived sequences were not detected.

| **Cell Sample** | **Protein** | **Confidence (%)** | **Sequence** | **Length (aa)** |
| --- | --- | --- | --- | --- |
| LCL 1 GFP | Calreticulin Human | 96.79 | KVHVIFNYK | 9 |
| LCL 1 GFP | Calreticulin Human | 97.17 | NPEYSPDPSIYAY | 13 |
| LCL 1 GFP | Calreticulin Human | 98.73 | NPEYSPDPSIYAYD | 14 |
| LCL 1 GFP | Calreticulin Human | 99.00 | NPEYSPDPSIYAYDN | 15 |
| LCL 1 mCALR-GFP | Calreticulin Human | 97.41 | NPEYSPDPSIYA | 12 |
| LCL 1 mCALR-GFP | Calreticulin Human | 97.79 | NPEYSPDPSIYAYD | 14 |
| LCL 1 mCALR-GFP | Calreticulin Human | 99.00 | NPEYSPDPSIYAYDN | 15 |
| LCL 1 mCALR-GFP | Calreticulin Human | 99.00 | DPPLIALDKDAPLR | 14 |
| LCL 2 GFP | Enhanced green fluorescent protein | 99.00 | ITLGMDELYK | 10 |
| LCL 2 GFP | Enhanced green fluorescent protein | 99.00 | SAMPEGYVQER | 11 |
| LCL 2 GFP | Enhanced green fluorescent protein | 99.00 | VELDGDVNGHKF | 12 |
| LCL 2 GFP | Enhanced green fluorescent protein | 99.00 | AGITLGMDELYK | 12 |
| LCL 2 mCALR-GFP | Enhanced green fluorescent protein | 99.00 | ITLGMDELYK | 10 |
| LCL 2 mCALR-GFP | Enhanced green fluorescent protein | 99.00 | SAMPEGYVQER | 11 |
| LCL 1 GFP | Enhanced green fluorescent protein | 99.00 | ITLGMDELYK | 10 |
| LCL 1 GFP | Enhanced green fluorescent protein | 96.88 | VSGEGEGDATY | 11 |
| LCL 1 GFP | Enhanced green fluorescent protein | 99.00 | TIFFKDDGNYK | 11 |
