# Supplementary material for: Isolation of T cell receptors targeting recurrent neoantigens in hematological malignancies
Source: J Immunother Cancer. 2018 Jul 13;6:70. doi: 10.1186/s40425-018-0386-y (PMC6044029; doi:10.1186/s40425-018-0386-y)
Supplement: Supplementary file 3 — Myeloproliferative neoplasm patient cohort. (DOCX 16 kb) [file 40425_2018_386_MOESM3_ESM.docx]

Additional file 3

**Myeloproliferative neoplasm patient cohort:** MPN patients expressing mutations in CALR exon 9 were identified by Sanger sequencing. HLA-A*03:01 or HLA-B*07:02 positive patients were determined by PCR. As negative controls for ex vivo mCALR multimer staining, a HLA-A*03:01^-^ HLA-B*07:02^-^ patient (MPN 016) and a JAK2 V617F +ve patient (assumed to be CALR exon 9 mutation -ve) (MPN 017) were included. ET= essential thrombocythaemia.

| **Patient ID** | **Disease** | **Gender** | **Treatment** | **Age** | **HLA** | **Mutation** |
| --- | --- | --- | --- | --- | --- | --- |
| MPN 006 | ET | Female | Hydroxycarbamide | 75 | HLA-A*03:01^+^  HLA-B*07:02^-^ | JAK2 V617F -ve |
| MPN 008 | ET | Female | Hydroxycarbamide | 48 | HLA-A*03:01^+^  HLA-B*07:02^+^ | JAK2 V617F -ve;  CALR: c.1154_1155insTTGTC;pLys385Asnfs*47 |
| MPN 009 | ET | Male | Hydroxycarbamide | 66 | HLA-A*03:01^-^  HLA-B*07:02^-^ | JAK2 V617F -ve;  CALR: c.1154_1155insTTGTC;pLys385Asnfs*47 |
| MPN 010 | ET | Female | Hydroxycarbamide  Anagrelide  Peginterferon alfa-2a | 44 | HLA-A*03:01^+^  HLA-B*07:02^-^ | JAK2 V617F -ve;  CALR: c.1154_1155insTTGTC;pLys385Asnfs*47 |
| MPN 013 | ET | Female | Hydroxycarbamide  Anagrelide  Ruxolitinib  Peginterferon alfa-2a | 56 | HLA-A*03:01^+^  HLA-B*07:02^+^ | JAK2 V617F -ve;  CALR: c.1154_1155insTTGTC;pLys385Asnfs*47 |
| MPN 016 | ET | Male | Hydroxycarbamide | 50 | HLA-A*03:01^-^  HLA-B*07:02^-^ | JAK2 V617F –ve;  CALR: c.1154-1155insTTGTC;p.Lys385Ansfs*47 |
| MPN 017 | ET | Male | Hydroxycarbamide | 67 | HLA-A*03:01^+^  HLA-B*07:02^+^ | JAK2 V617F +ve |
