# Supplementary material for: Isolation of T cell receptors targeting recurrent neoantigens in hematological malignancies
Source: J Immunother Cancer. 2018 Jul 13;6:70. doi: 10.1186/s40425-018-0386-y (PMC6044029; doi:10.1186/s40425-018-0386-y)
Supplement: Supplementary file 4 — Neoepitope-specific T cell enrichment work flow. (DOCX 233 kb) [file 40425_2018_386_MOESM4_ESM.docx]

Additional file 4

**Neoepitope-specific T cell enrichment work flow:** A protocol was designed for the isolation of mutant neoepitope-specific CD8^+^ T cells using MHC class I multimers and cell-sorting technologies from the blood of healthy donors. Neoepitope-specific T cells were cloned by single-cell sorting and tested for their ability to recognize mutant target cells. Subsequently, neoepitope-specific TCRαβ genes were sequenced and transferred into healthy donor T cells by retroviral-mediated TCR gene transfer.

**
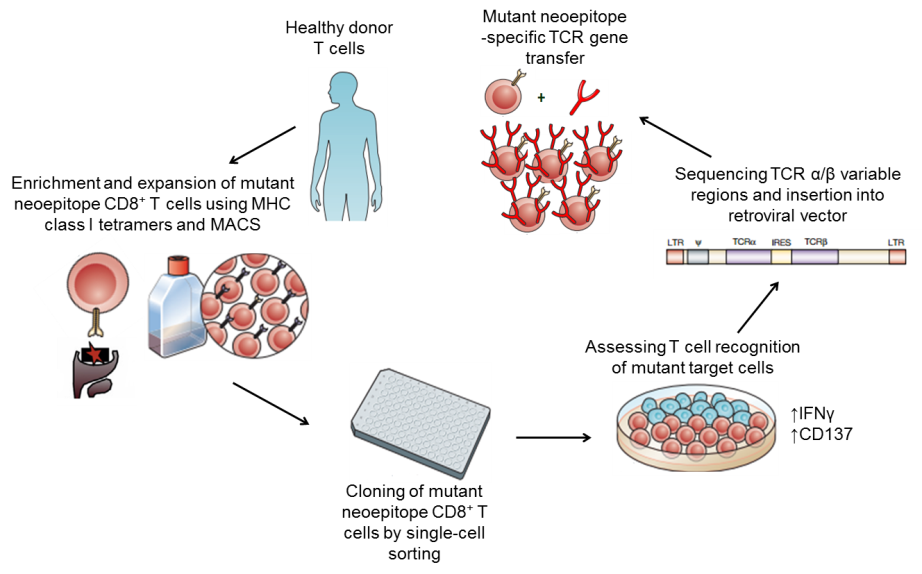
**
