# Supplementary material for: Isolation of T cell receptors targeting recurrent neoantigens in hematological malignancies
Source: J Immunother Cancer. 2018 Jul 13;6:70. doi: 10.1186/s40425-018-0386-y (PMC6044029; doi:10.1186/s40425-018-0386-y)
Supplement: Supplementary file 5 — Detection of cancer-testes antigen T cell responses in the healthy donor T cell repertoire. (DOCX 1988 kb) [file 40425_2018_386_MOESM5_ESM.docx]

Additional file 5

**Detection of CT antigen T cell responses in the healthy T cell repertoire.** (A) Following enrichment and expansion of CT antigen-specific T cells from healthy PBMCs, CT antigen CD8^+^ T cell responses were detected using PE and APC conjugated HLA-A*02:01 multimers. T cells were stained with a pool of 11 CT antigen multimers, as well as individual CT antigen multimers. Flow cytometry plots are gated on live CD8^+^ cells. n=1. (B) Following the detection of a LAGE-1 multimer^+^ T cell population, a LAGE-1 multimer^+^ T cell clone was successfully isolated.


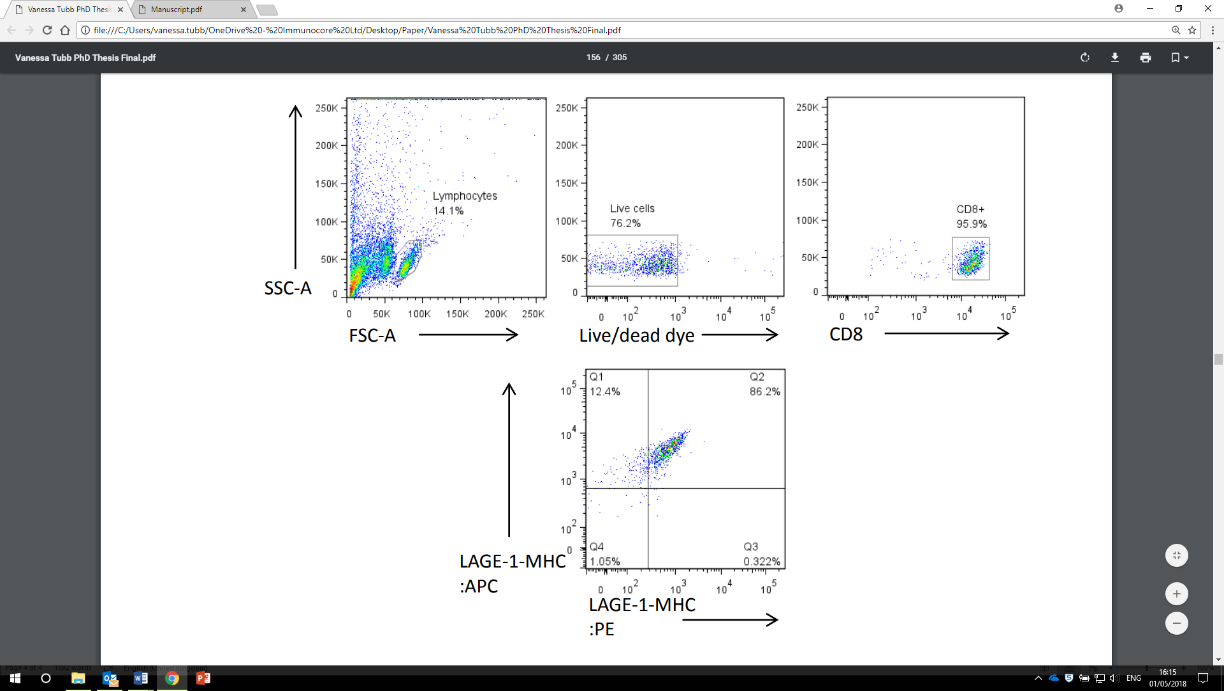


**B**


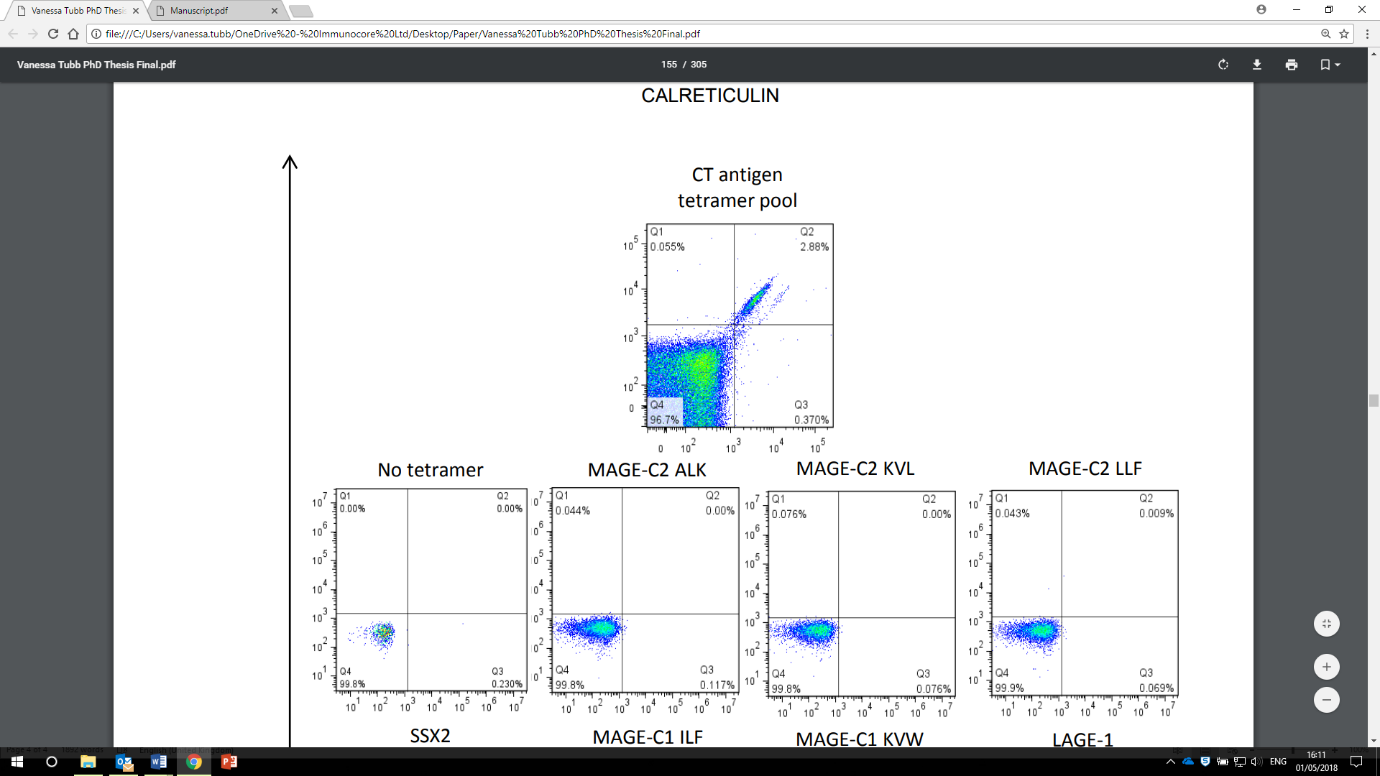

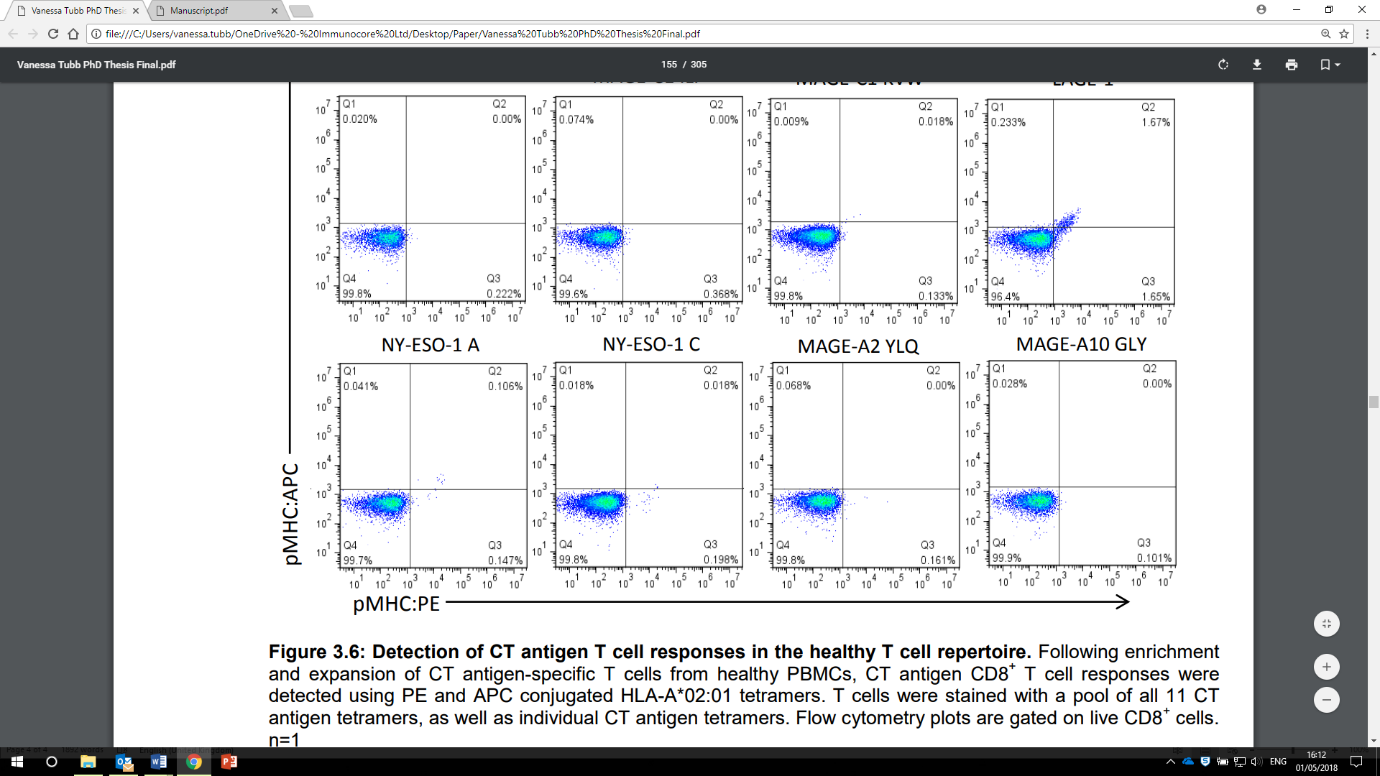


**A**
