# Supplementary material for: Isolation of T cell receptors targeting recurrent neoantigens in hematological malignancies
Source: J Immunother Cancer. 2018 Jul 13;6:70. doi: 10.1186/s40425-018-0386-y (PMC6044029; doi:10.1186/s40425-018-0386-y)
Supplement: Supplementary file 6 — mCALR-specific TCR gene rearrangments. (DOCX 1896 kb) [file 40425_2018_386_MOESM6_ESM.docx]

Additional file 6

**mCALR-specific TCR gene rearrangements:** The four most reactive mCALR T cell clones (CALR-RMR clone 4, CALR-RMR clone 20, CALRp7 clone 2 and CALRp7 clone 3) were chosen for TCRαβ variable gene sequencing using TCR gene capture technology ([1](#_ENREF_2)). Circular plots represent TCRα and β loci chromosomal rearrangements. Chromosomal positions of TCR variable elements are shown in blue, and TCR joining elements are shown in red.

CALRp7 clone 3

CALRp7 clone 2

CALR-RMR clone 20

CALR-RMR clone 4

TCRβ chain
Chromosome 7

TCRα chain
Chromosome 14

References

1. Linnemann C, Heemskerk B, Kvistborg P, Kluin RJC, Bolotin DA, Chen X, et al. High-throughput identification of antigen-specific TCRs by TCR gene capture. Nature Medicine. 1534-2013;19(11):1534-43.
