# Supplementary material for: Isolation of T cell receptors targeting recurrent neoantigens in hematological malignancies
Source: J Immunother Cancer. 2018 Jul 13;6:70. doi: 10.1186/s40425-018-0386-y (PMC6044029; doi:10.1186/s40425-018-0386-y)
Supplement: Supplementary file 7 — Engineering of mCALR-expressing target cells. (DOCX 488 kb) [file 40425_2018_386_MOESM7_ESM.docx]

Additional file 7

**Engineering of mCALR-expressing target cells:** (A) HLA-A*03:01^+^ HLA-B*07:02^+^ LCLs were retrovirally transduced with a mCALR-GFP mini gene. (B) To confirm mCALR gene expression RNA was isolated from mCALR-GFP^+^ LCLs, DNase treated, and converted to cDNA. mCALR expression was determined by mCALR PCR. No reverse trancriptase (RT) controls were included to exclude any mCALR amplification from contaminating DNA. The pmx-mCALR-IRES-GFP DNA vector was used as a positive control. mCALR product = 113bps. (n=1) (C) MARIMO cells naturally expressing a frameshift mutation in CALR exon 9 were retrovirally transduced with HLA-A*03:01-GFP and HLA-B*07:02-GFP.

**A**


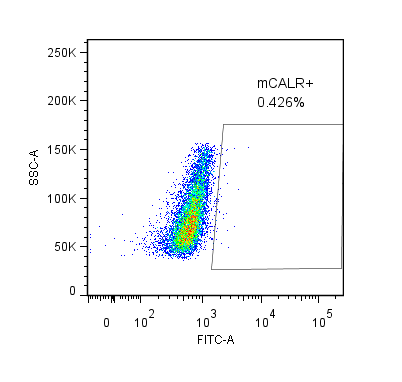

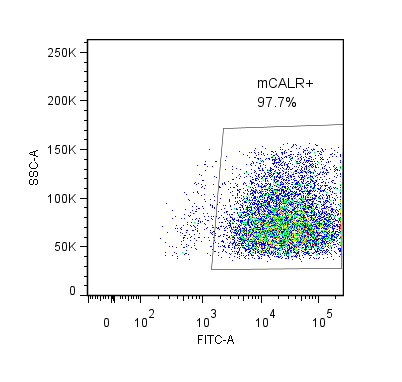


mCALR-GFP-transduced


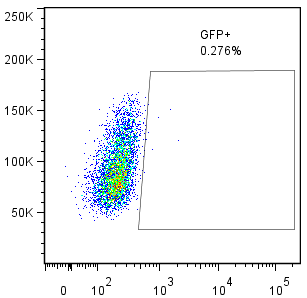

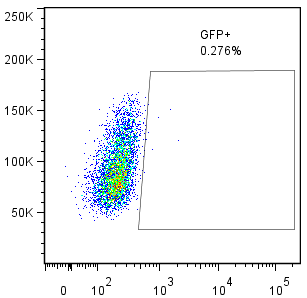


HLA-A*03:01^+^

HLA-B*07:02^+^

LCL

Mock-transduced


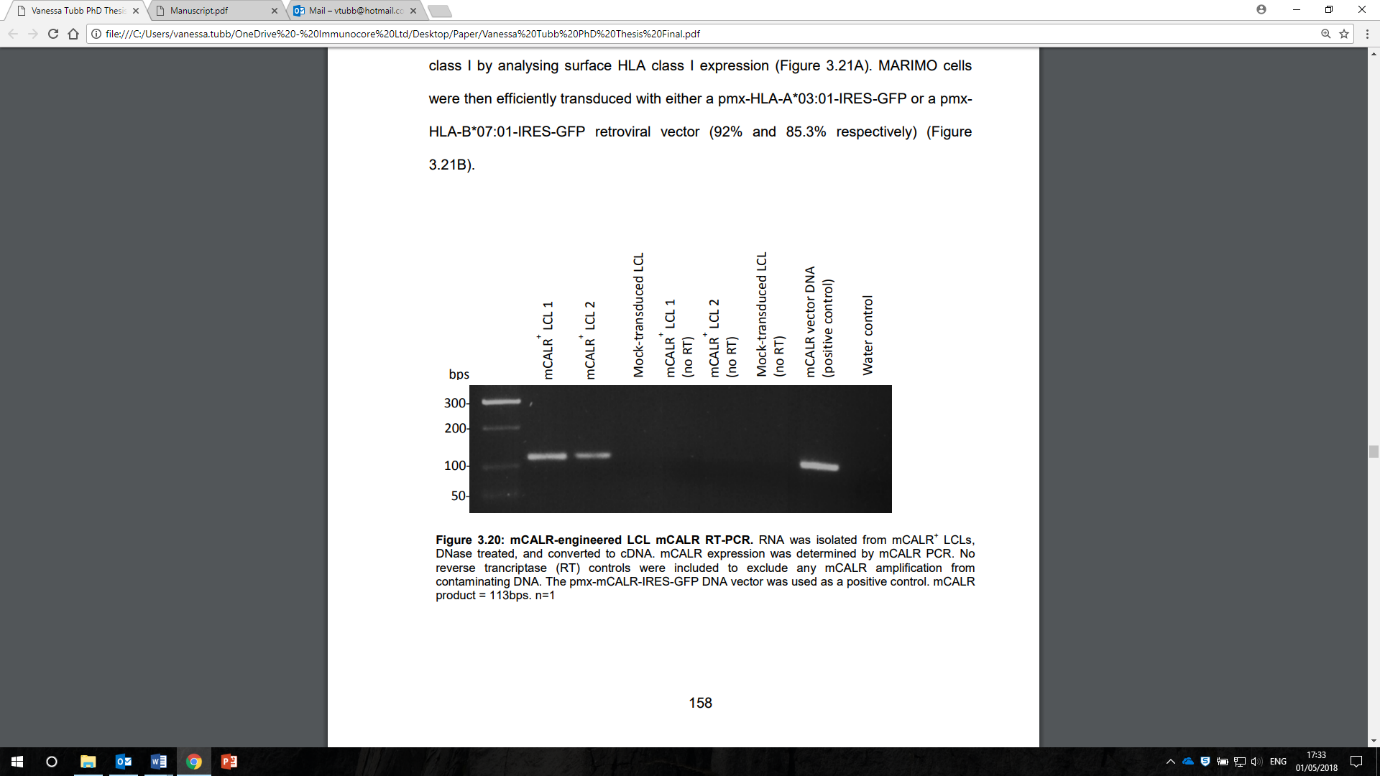


**B**

HLA-B7-GFP-transduced

HLA-A3-GFP-transduced

Mock-transduced


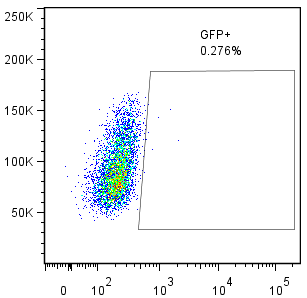

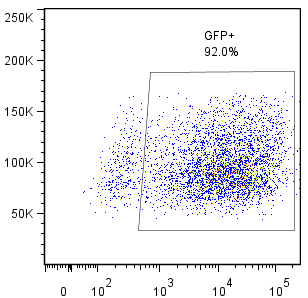

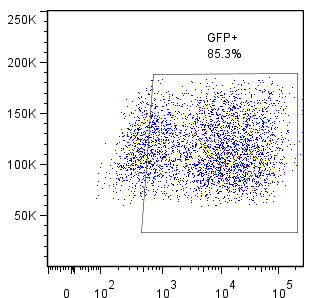


MARIMO

**C**
