# Supplementary material for: Isolation of T cell receptors targeting recurrent neoantigens in hematological malignancies
Source: J Immunother Cancer. 2018 Jul 13;6:70. doi: 10.1186/s40425-018-0386-y (PMC6044029; doi:10.1186/s40425-018-0386-y)
Supplement: Supplementary file 8 — Engineering of mFBXW7-expressing target cells. (DOCX 409 kb) [file 40425_2018_386_MOESM8_ESM.docx]

Additional file 8

**Engineering mFBXW7-expressing target cells:** (A) HLA-A*11:01^+^ LCLs were retrovirally-transduced with an mFBXW7-GFP mini gene and flow sorted by GFP expression. (B) GFP-sorted LCLs were subjected to mFBXW7 RT-PCR to detect mFBXW7 RNA expression. No RT controls were included to rule out DNA contamination. mFBXW7 length = 212bps. (n=2) (C) FBXW7 R465H^+^ T-ALL cell lines (CML-T1 and RPMI8402) were retrovirally transduced with a HLA-A*11:01-GFP vector and flow sorted by GFP expression.

Mock-transduced LCL


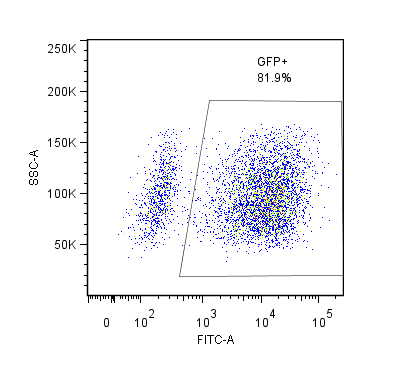

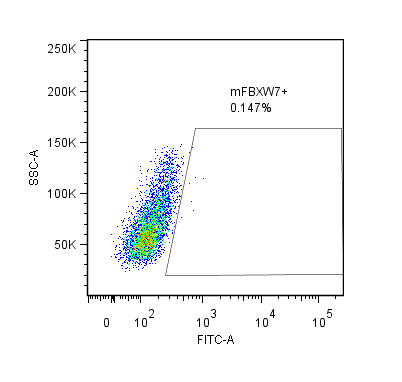


mFBXW7-GFP

transduced LCL


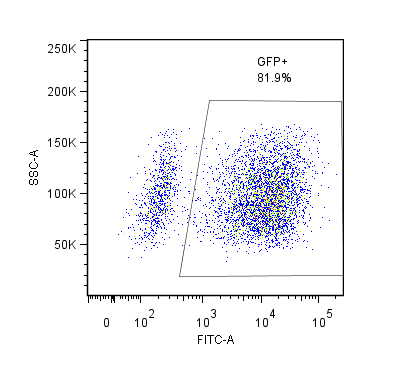


**A**


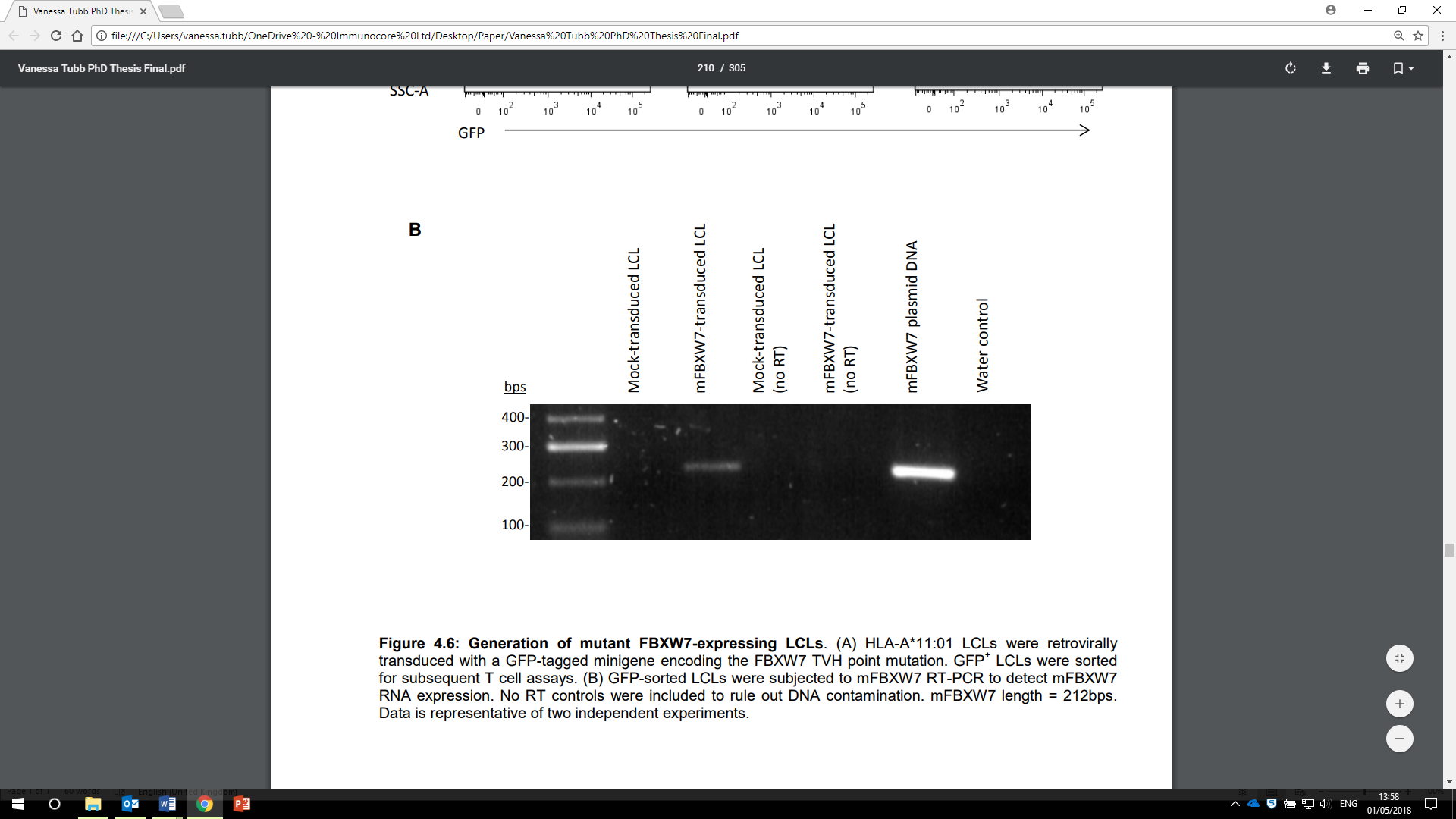


**B**


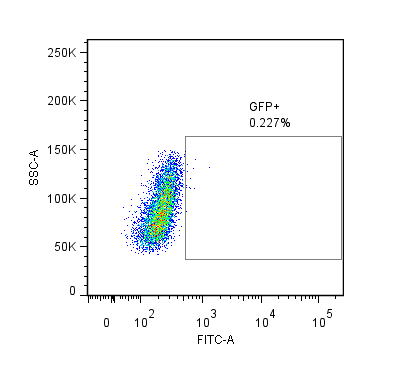

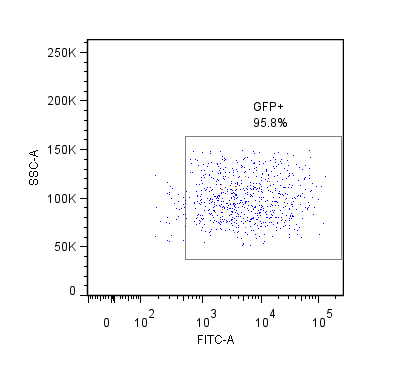

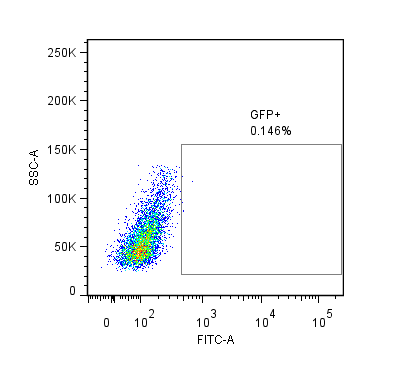

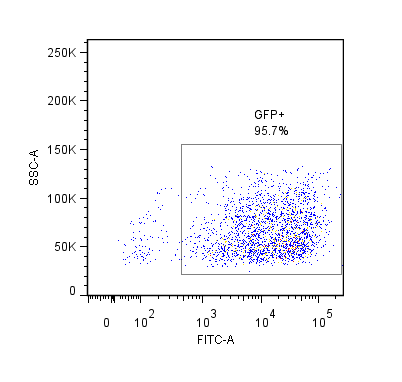


Mock-transduced

HLA-A*11:01-GFP

transduced

RPMI 8402 cells

CML-T1 cells

**C**
